# Activation of basal forebrain-to-lateral habenula circuitry drives reflexive aversion and suppresses feeding behavior

**DOI:** 10.1038/s41598-022-26306-8

**Published:** 2022-12-21

**Authors:** Jessica L. Swanson, Joshua Ortiz-Guzman, Snigdha Srivastava, Pey-Shyuan Chin, Sean W. Dooling, Elizabeth Hanson Moss, Mikhail Y. Kochukov, Patrick J. Hunt, Jay M. Patel, Brandon T. Pekarek, Qingchun Tong, Benjamin R. Arenkiel

**Affiliations:** 1 Department of Molecular and Human Genetics, Baylor College of Medicine, Houston, TX USA; 2 Jan and Dan Duncan Neurological Research Institute, Texas Children’s Hospital, Houston, TX USA; 3 Medical Scientist Training Program, Baylor College of Medicine, Houston, TX USA; 4 Department of Neuroscience, Baylor College of Medicine, Houston, TX USA; 5 Center for Metabolic and Degenerative Disease, The University of Texas Health Science Center at Houston, Houston, TX USA

**Keywords:** Central nervous system, Olfactory system, Biological techniques, Genetics, Neuroscience

## Abstract

Environmental cues and internal states such as mood, reward, or aversion directly influence feeding behaviors beyond homeostatic necessity. The hypothalamus has been extensively investigated for its role in homeostatic feeding. However, many of the neural circuits that drive more complex, non-homeostatic feeding that integrate valence and sensory cues (such as taste and smell) remain unknown. Here, we describe a basal forebrain (BF)-to-lateral habenula (LHb) circuit that directly modulates non-homeostatic feeding behavior. Using viral-mediated circuit mapping, we identified a population of glutamatergic neurons within the BF that project to the LHb, which responds to diverse sensory cues, including aversive and food-related odors. Optogenetic activation of BF-to-LHb circuitry drives robust, reflexive-like aversion. Furthermore, activation of this circuitry suppresses the drive to eat in a fasted state. Together, these data reveal a role of basal forebrain glutamatergic neurons in modulating LHb-associated aversion and feeding behaviors by sensing environmental cues.

## Introduction

Feeding is an appetitive behavior that is essential for the survival of all animals. Homeostatic feeding, or feeding to meet caloric requirements, consists of balancing caloric output with caloric intake to maintain proper weight and metabolic health. However, this is only one component of feeding behavior. Environmental cues (such as taste and smell), mood, reward, and aversion all affect feeding and can drive food consumption beyond or below normal healthy caloric requirements^1–3^. In contrast to homeostatic feeding, these non-homeostatic feeding mechanisms have evolved to make organisms adaptable to a changing environment, in which food sources may be unreliable. However, when food is easily accessible, these mechanisms can become maladaptive.

A classic example of non-homeostatic feeding is reward-based hedonic behavior that drives an animal to consume food beyond caloric necessity. Reciprocally, aversive food cues and/or threatening stimuli may prevent food intake even in a fasted state. For example, cues that indicate spoiled food or a nearby predator may drive overriding avoidance or escape behavior, respectively, to ensure survival. While it is generally appreciated that the hypothalamus regulates key aspects of homeostatic feeding^4–8^, and that homeostasis, reward, and aversion pathways converge to govern feeding^9–11^, the circuits, neuronal constituents, and patterns of functional connectivity that mediate non-homeostatic feeding behavior remain largely unknown.

We and others have recently identified the basal forebrain as a circuit node that directly impacts non-homeostatic feeding^12–14^. Notably, when excitatory, glutamatergic neurons of the BF were genetically targeted for chronic activation, mice exhibited severe, lethal hypophagia. This feeding suppression was accompanied by aversion to food and food-related stimuli. Glutamatergic BF projections to the lateral hypothalamic area (LHA) were identified as partially responsible for both the observed hypophagia and aversion, however, direct activation of glutamatergic BF terminals within the LHA did not fully phenocopy the food-associated aversion displayed by BF cell body activation, suggesting that other downstream targets of the BF contribute to the observed food-related aversion^12^.

Through viral-mediated anterograde projection mapping, we found that glutamatergic neurons of the BF also project to the Lateral Habenula (LHb), a prominent aversion center within the brain^3,15^, and that the LHb receives sensory information from the BF. Further, when BF-to LHb projections are activated, this circuit drives a potent, reflex-like aversion that disrupts memory. This circuitry suppresses the homeostatic drive to eat without affecting appetite. Together, these data identify a brain circuit that links the glutamatergic basal forebrain to the LHb to directly modulate feeding independent of homeostatic state.

## Results

### Glutamatergic basal forebrain neurons are functionally connected to the lateral habenula

**Figure 1:**
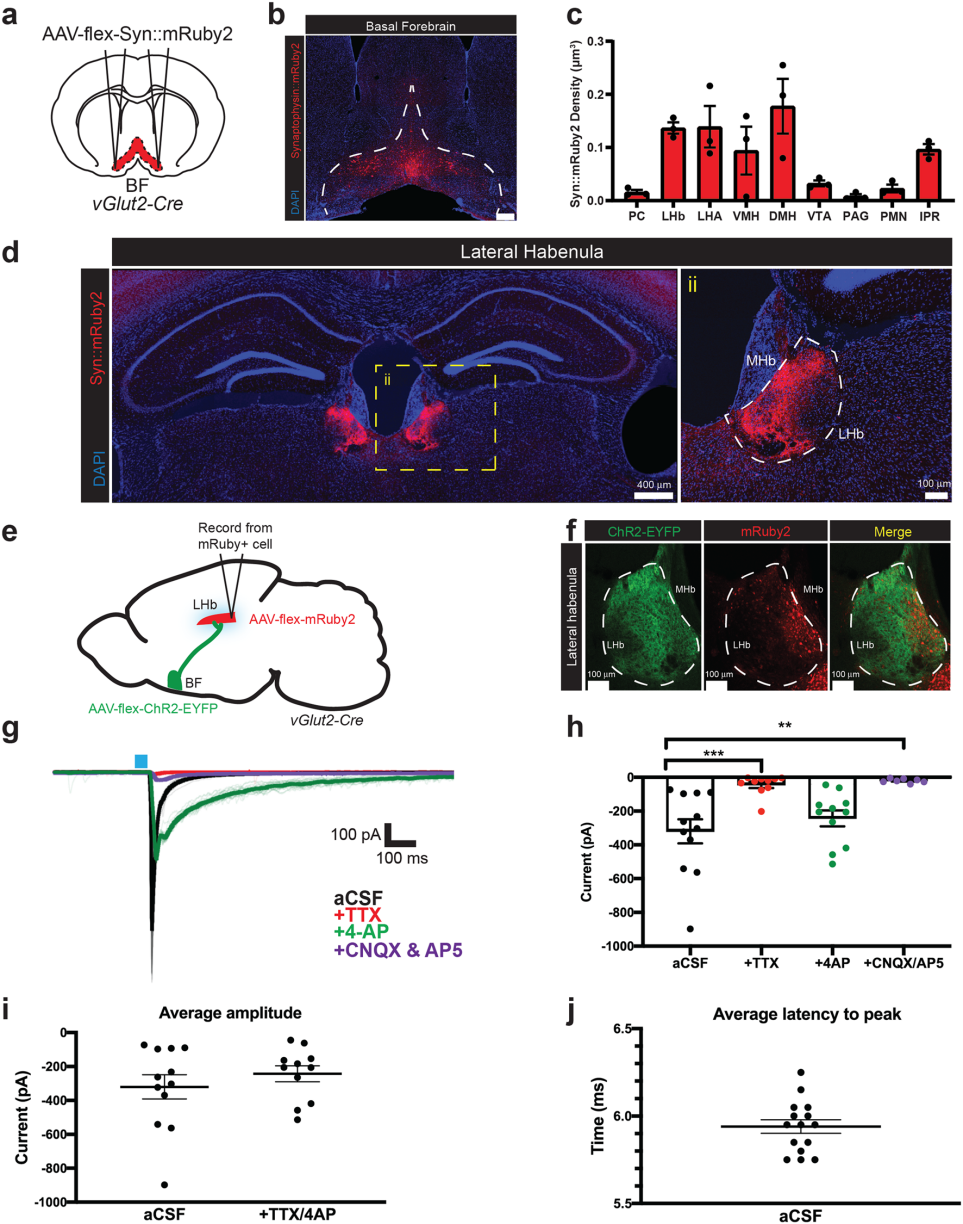
Glutamatergic BF neurons robustly innervate the LHb and are functionally connected to glutamatergic LHb neurons. (**a**) Experimental setup for anterograde tracing from vGlut2^BF^ cells. (**b**) Representative viral targeting of Cre-dependent Synaptophysin::mRuby2 to the basal forebrain (BF, Bregma 0.62). Scale bar = 300 μm. (**c**) Quantification of different brain regions receiving glutamatergic BF input. Quantification calculated as the density of Syn::mRuby2+ terminals/volume of the ROI. PC, piriform cortex; LHb, lateral habenula; LHA, lateral hypothalamic area; VMH, ventromedial hypothalamus; DMH, dorsomedial hypothalamus; VTA, ventral tegmental area; PAG, periaqueductal gray; PMN, premammillary nucleus; IPR, interpeduncular nucleus. (**d**) Synaptophysin::mRuby2 projection terminals in the lateral habenula (LHb) (Bregma − 1.82). ii) Zoomed-in inset. MHb, medial habenula. (**e**) Experimental setup of channelrhodopsin-assisted circuit mapping experiment, stimulating vGlut2+ BF terminals while recording from mRuby+/vGlut2+ cells in the LHb. (**f**) ChR2-EYFP fibers from the vGlut2^BF^ neurons terminating in the LHb. mRuby+ vGlut2^LHb^ cells in the LHb overlap with ChR2-expressing BF projections. (**g**) Representative ex vivo electrophysiological trace from mRuby+ cell in the LHb. CSF, cerebral spinal fluid (control). TTX (1 μM), 4-AP (0.5 μM), and CNQX/AP5 (10 μM/ 50 μM) added sequentially. (**h**) Current measurements (pA) of mRuby+ LHb cells after optogenetic stimulation of BF terminals in (1) aCSF (− 320.20 ± 71.30 pA), (2) + TTX (− 44.20 ± 19.20 pA), (3) + 4AP (− 243.10 ± 47.08 pA) and (4) + CNQX/AP5 (− 20.43 ± 4.64 pA). Error bars represent SEM. Statistically compared using a One-Way ANOVA comparing all groups to the aCSF control group. aCSF vs. TTX: *p* = 0.0010, aCSF vs. + 4AP: *p* = 0.5496, aCSF vs. + CNQX/AP5: *p* = 0.0013. n = 12 for aCSF, n = 10 for TTX, n = 11 for 4AP, and n = 7 for CNQX/AP5. (**i**) Average amplitude of mRuby+ LHb cells in aCSF and with the addition of both TTX and 4AP. Error bars represent SEM. n = 12 and 11, respectively. aCSF = − 320.20 ± 71.30 pA, + TTX/4AP = − 243.10 ± 47.08 pA. Statistically compared using an unpaired t-test, *p* = 0.3859. (**j**) Average latency to 10% of the max current of mRuby+ LHb cells upon photostimulation of BF terminals (5.940 ± 0.04 ms). Error bars represent SEM. n = 15.

To determine how vGlut2^BF^ neurons may drive the cessation of feeding through avoidance/aversion circuits, we first sought to identify downstream effector targets implicated in aversive behaviors. Towards this, we performed anterograde projection mapping by stereotaxically injecting a Cre-dependent Synaptophysin::mRuby2 adeno-associated virus (rAAV-Ef1α-flex-Synaptophysin::mRuby2) into the basal forebrains of vGlut2-Cre^+/−^ mice, allowing for identification of presynaptic BF terminals and their presumptive downstream targets (Fig. 1a, b). We observed numerous termini in previously described regions with known glutamatergic BF input, including the lateral hypothalamic area (LHA), which we investigated previously (Fig. 1c, Supp. Fig. 1^12^). Notably, the lateral habenula (LHb) was robustly innervated by BF projections (Fig. 1d). Given that the lateral habenula is known to drive aversive behaviors^3,15^, we investigated vGlut2^BF^-to-LHb (vGlut2^BF→LHb^) connectivity as a candidate circuit to mediate aversion and feeding suppression.

To support the anatomical tracing data, we next tested whether the basal forebrain and lateral habenula are functionally connected. Since previous studies showed that the LHb contains mostly vGlut2+ cells^16^, we injected an AAV expressing Cre-dependent Channelrhodopsin (ChR2) into the BF (rAAV-Ef1α-flex-hChR2(H134R)-EYFP-WPRE-pA) and a Cre-dependent mRuby2 reporter virus into the LHb (rAAV-Ef1α-flex-mRuby2) of vGlut2-Cre^+/−^ animals (Fig. 1e, f). Then, we selectively photostimulated vGlut2^BF^ neurons that project to the LHb, while making visually guided whole cell recordings from mRuby+ vGlut2+ cells within the LHb. In LHb target neurons recorded, we observed robust depolarization with vGlut2^BF^ photostimulation with an average amplitude of − 320.20 ± 71.30 pA (Fig. 1g–i). Additionally, the average latency from the end of a 473 nm light pulse to 10% max current was 5.94 ± 0.039 ms, suggesting a monosynaptic connection (Fig. 1j). Moreover, performing recordings in the presence of TTX and 4-AP indicated that the vGlut2^BF→LHb^ connections were monosynaptic (Fig. 1g–i). This response was abolished in the presence of the AMPA and NMDA receptor antagonists CNQX and APV, indicating a glutamatergic response (Fig. 1g, h). Collectively, these data show that vGlut2^LHb^ cells receive robust innervation from vGlut2^BF^ neurons, and that these nodes are monosynaptically connected.

### The lateral habenula receives diverse olfactory sensory cues from the basal forebrain

**Figure 2:**
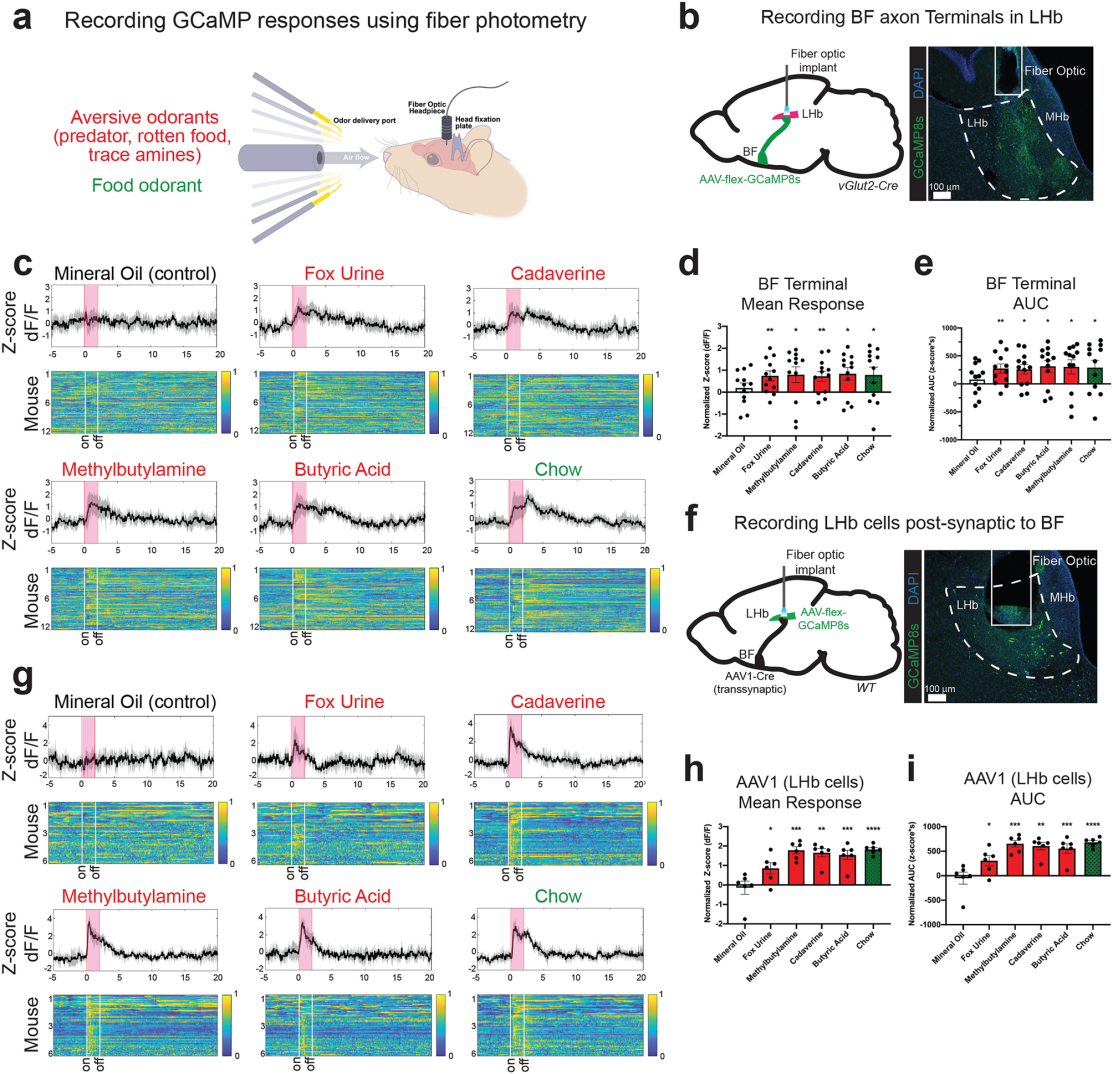
Glutamatergic BF-to-LHb projections and LHb cells receiving BF input respond to aversive and food-related sensory stimuli. (**a**) Experimental setup for fiber photometry recordings. (**b**) Experimental setup for recording vGlut2^BF^ axon terminals in the LHb and representative image of fiber optic targeting (Bregma − 1.58). (**c**) Average z-score dF/F traces of vGlut2^BF→LHb^ axon terminals (in black) across biological replicates (n = 12). Grey outline represents 95% CI. Pink box represents 2 s odor delivery. Heat map shows each presentation of a given odor (10 replicates per odor) across all 12 mice. Yellow = 1 (maximum activity), dark blue = 0 (minimum activity). Heat map generated by MATLAB (version R2019a; https://www.mathworks.com/products/matlab.html). (**d**) Mean odor response of BF Terminals recorded using fiber photometry. Error bars represent SEM. n = 12. Mean z-score dF/F odor responses: Mineral oil = 0.19 ± 0.22 (*p* = 0.41), Fox urine = 0.74 ± 0.22 (*p* = 0.0070), Chow = 0.79 ± 0.35 (*p* = 0.048), Methylbutylamine = 0.80 ± 0.36 (*p* = 0.047), Cadaverine = 0.73 ± 0.23 (*p* = 0.0087), Butyric acid = 0.84 ± 0.28 (*p* = 0.013). (**e**) Mean odor response area under the curve (AUC) of BF Terminals recorded using fiber photometry. Error bars represent SEM. n = 12. Mean AUC of z-score dF/F odor responses: Mineral oil = 78.61 ± 80.24 (*p* = 0.35), Fox urine = 273.0 ± 82.50 (*p* = 0.0070), Chow = 289.1 ± 131.1 (*p* = 0.049), Methylbutylamine = 302.0 ± 127.6 (*p* = 0.037), Cadaverine = 256.9 ± 85.95 (*p* = 0.012), Butyric acid = 312.7 ± 103.5 (*p* = 0.012). (**f**) Experimental setup for fiber photometry recordings of LHb cells receiving BF input using AAV1-Cre and representative image of fiber optic targeting (Bregma − 1.58). (**g**) Average z-score dF/F traces of LHb cells receiving BF input (in black) across biological replicates (n = 6). Grey outline represents 95% CI. Pink box represents 2 s odor delivery. Heat map shows each presentation of a given odor (10 replicates) across all 6 mice. Yellow = 1 (maximum activity), dark blue = 0 (minimum activity). Heat map generated by MATLAB (version R2019a; https://www.mathworks.com/products/matlab.html). (**h**) Mean odor response of LHb cells receiving BF input recorded using fiber photometry. Error bars represent SEM. n = 6. Mean z-score dF/F odor responses: Mineral oil: − 0.14 ± 0.34 (*p* = 0.69), Fox urine = 0.85 ± 0.29 (*p* = 0.033), Chow = 1.83 ± 0.10 (*p* =  < 0.0001), Methylbutylamine = 1.78 ± 0.18 (*p* = 0.0002), Cadaverine = 1.66 ± 0.21 (*p* = 0.0006), Butyric acid = 1.54 ± 0.24 (*p* = 0.0013). (**i**) Mean odor response area under the curve (AUC) of LHb cells receiving BF input recorded using fiber photometry. Error bars represent SEM. n = 6. Mean AUC of z-score dF/F odor responses: Mineral oil = − 51.10 ± 123.3 (*p* = 0.70), Fox urine = 306.0 ± 103.0 (*p* = 0.031), Chow = 680.9 ± 37.48 (*p* =  < 0.0001), Methylbutylamine = 652.9 ± 66.78 (*p* = 0.0002), Cadaverine = 604.0 ± 76.99 (*p* = 0.0005), Butyric acid = 553.7 ± 95.45 (*p* = 0.0021).

In addition to driving feeding suppression and aversion, vGlut2^BF^ neurons also respond to diverse sensory stimuli, including aversive and food-related olfactory cues^12,17,18^. Given that the BF and LHb are functionally connected, we next sought to determine if BF projections to the LHb or neurons within the LHb itself respond to sensory information. Due to the abundance and diversity of volatile odorants, and because certain odors are known to be innately aversive, appetitive, or rewarding to mice^19–21^, we presented olfactory stimuli while recording the neural activity of vGlut2^BF^ axon terminals within the LHb. For this, we targeted expression of Cre-dependent GCaMP8 (rAAV-Synapsin-flex-jGCaMP8s) to vGlut2^BF^ neurons and performed fiber photometry in the LHb while we presented either appetitive food-related^19,20^ or innately aversive odors (fox urine, rotten food odor, and trace amines^19,21^) using a previously described continuous-flow olfactometer (Fig. 2a, b, Supp. Fig. 2a^22^). Odorants were presented for 2 s each, in replicates of 10 with a randomized order, and 18 s between odor presentation.

Upon odor delivery, vGlut2^BF→LHb^ terminals responded to both aversive and food-related odors, but not to the mineral oil controls (Fig. 2c). Quantification of photometric recordings, including both the average odor response and average area under the curve (AUC) revealed that vGlut2^BF→LHb^ neurons were activated at similar levels by both food and innately aversive odors (Fig. 2d, e, Supp. Fig. 3). For example, comparing baseline subtracted z-score values of the average odor responses, vGlut2^BF→LHb^ terminals showed increased fluorescence by 0.80 ± 0.36 (*p* = 0.047) to the trace amine methylbutylamine, and increased by 0.79 ± 0.35 (*p* = 0.048) for chow odorant (Fig. 2d). VGlut2^BF^ projections to the LHb were less likely to respond to neutral odors and did not respond to the appetitive, palatable food odorant peanut butter (Supp. Fig. 4). Thus, vGlut2^BF^ projections to the LHb appear to respond to both aversive and appetitive sensory information. Given the broad responses of vGlut2^BF→LHb^ terminals, we next questioned to what extent neurons in the LHb receive and directly respond to this sensory information. Towards this, we stereotaxically targeted an anterograde, transsynaptic Cre (rAAV1-hSyn-Cre)^23^ to the BF of wildtype animals (C57BL/6NJ), while simultaneously injecting and implanting the LHb with Cre-dependent GCaMP8s (rAAV-Synapsin-flex-jGCaMP8s) and a fiber optic implant for photometric recordings (Fig. 2f, Supp. Fig. 2b). This allowed us to record from LHb target cells that receive BF synaptic input. Consistent with what we observed from photometry responses in vGlut2^BF^ terminals, we noted robust odor responses to both aversive and food-related odors in LHb neurons (Fig. 2g–i, Supp. Fig. 5). For example, comparing baseline subtracted z-score values of the average odor responses, LHb cells targeted by AAV1-Cre in the BF showed increased fluorescence by 1.78 ± 0.18 (*p* = 0.0002) to the trace amine methylbutylamine, and by 1.83 ± 0.10 (*p* =  < 0.0001) to chow odorant (Fig. 2h). LHb cells receiving BF input also appeared to respond to several neutral odors and to the appetitive palatable food odorant peanut butter (Supp. Fig. 6). Thus, vGlut2^BF→LHb^ projections relay a wide array of sensory information, including both appetitive/food-related and aversive cues to the LHb, which also responds to diverse olfactory stimuli.

### Lateral habenula projecting glutamatergic basal forebrain neurons suppress feeding behavior

**Figure 3:**
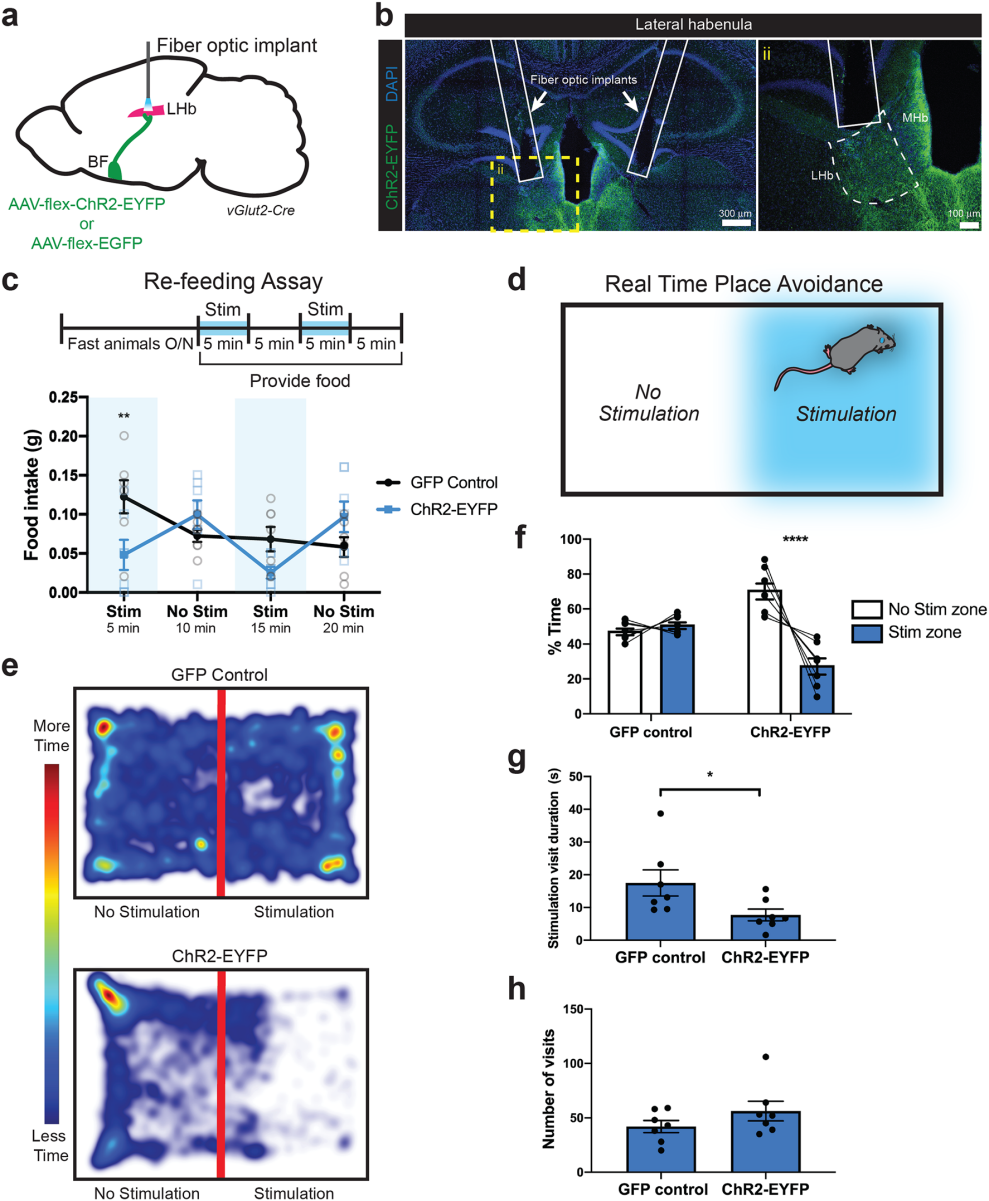
Activation of lateral habenula-projecting glutamatergic basal forebrain neurons reduces food intake and drives aversion. (**a**) Experimental setup for in vivo optogenetic behavior, in which vGlut2^BF^ terminals in the LHb are activated. (**b**) Representative images showing fiber optic implant targeting BF terminals in the LHb. Images taken at Bregma − 1.46. ii) Zoomed-in inset. (**c**) Experimental timeline for re-feeding assay with and without optogenetic stimulation of vGlut2^BF→LHb^ cells. Average food intake (g) of chow measured in 5 min stim/no stim intervals throughout the duration of a 20 min re-feeding experiment. Solid symbols represent averaged values, while hollow/transparent symbols represent individual values biological replicates. Statistical significance calculated using repeated measures two-way ANOVA with Sidak correction for multiple comparisons. Error bars represent SEM. n = 7. At 5 min stim time period: GFP controls = 0.12 ± 0.021 g, ChR2 animals = 0.05 ± 0.019 g, *p* = 0.0078. At 10 min non-stim time period: GFP controls = 0.07 ± 0.008 g, ChR2 = 0.100 ± 0.018 g, *p* = 0.6608. At 15 min stim time period: GFP controls = 0.07 ± 0.016 g, CHR2 = 0.03 ± 0.008 g, *p* = 0.2345. At 20 min non-stim time period: GFP controls = 0.06 ± 0.013 g, ChR2 = 0.097 ± 0.020 g, *p* = 0.3297. (**d**) Experimental setup for real-time place avoidance assay with optogenetic stimulation. (**e**) Heat maps showing movement of representative GFP control and ChR2-EYFP mice during the real-time place avoidance assay in which mice were stimulated on the right side of the chamber. Heat maps generated using Noldus Noldus EthoVision (XT 16; https://www.noldus.com/ethovision-xt) software. (**f**) Average percent time GFP controls and ChR2-EYFP expressing animals spent in non-stimulation or stimulation sides of the arena during a 20 min experiment. Statistical significance determined using Binomial test for proportion with Bonferroni correction; null hypothesis = 50%. Error bars represent SEM. n = 7. For GFP controls, they spent 47.68 ± 1.98% of their time in the non-stim side and 51.32 ± 1.97% in the stim side, *p* = 0.7644. For ChR2: 71.07 ± 4.70% time in non-stim side, 27.84 ± 4.82% in stim side, *p* < 0.0001. (**g**) Average duration of each visit to the stimulation side of the chamber. Statistical significance determined using unpaired, two-tailed t-test. GFP controls = 17.50 ± 3.98 s, ChR2 = 7.71 ± 1.79 s, *p* = 0.0447. n = 7. (**h**) Number of visits to the stimulation zone. Statistical significance determined using unpaired, two-tailed t-test. GFP controls = 42.00 ± 5.52 visits, ChR2 = 56.29 ± 9.05 visits, *p* = 0.2026 (not significant). n = 7.

We have identified the glutamatergic BF as a major node in suppressing food intake and driving aversion, and have shown that vGlut2^BF^ neurons send robust projections to the LHb. Thus, we sought to determine if vGlut2^BF→LHb^ connectivity modulates feeding. Towards this, we stereotaxically targeted an AAV expressing Cre-dependent Channelrhodopsin2-EYFP (rAAV-Ef1α-flex-hChR2(H134R)-EYFP)^24^, or Cre-dependent GFP as a control (rAAV-EF1α-flex-GFP) to the BF of vGlut2-Cre^+/−^ animals. ChR2-EYFP, which is membrane bound, robustly labeled BF→LHb projections in a similar manner as synaptophysin::mRuby2 labeling (Fig. 1d). However, given that ChR2 is membrane localized, fibers of passage from the BF to the LHb were also visualized passing through the thalamus. Fiber optics were implanted over the LHb to selectively stimulate ChR2-expressing vGlut2^BF^ terminals in the LHb (Fig. 3a, b, Supp. Fig. 7).

To test the effect of vGlut2^BF→LHb^ activation on feeding behavior, mice were fasted overnight and then stimulated with 470 nm light at 20 Hz (the maximal firing rate of vGlut2^BF^ neurons^25^) with 5 ms pulses for 5 min, followed by 5 min off periods while presented with food. Food consumption was measured every 5 min for a total of 20 min (Fig. 3c). ChR2-expressing mice drastically reduced their food intake by ~ 50% during periods of photostimulation, but consumed as much food as control animals during periods without photostimulation (Fig. 3c; ChR2-EYFP mice consumed 0.05 ± 0.02 g chow, while GFP controls consumed 0.12 ± 0.02 g in the initial stimulation bout (*p* = 0.0078)). Thus, selective activation of vGlut2^BF→LHb^ terminals transiently and significantly prevented food intake in fasted animals, but immediately upon cessation of photostimulation, normal unabated feeding behavior resumed.

### Lateral habenula projecting glutamatergic basal forebrain neurons drive aversive behaviors

Since the glutamatergic BF has been shown to mediate aversion^12,26^, we next asked whether optogenetic activation of vGlut2^BF→LHb^ terminals mediate aversive behavior. To test this, we subjected LHb-implanted optogenetic mice (Fig. 3a, b) to a real-time place avoidance assay, in which mice were photostimulated (20 Hz, 5 ms pulses) in one half of an unmarked arena (Fig. 3d). Mice were video-recorded, and the time spent in either half of the arena was analyzed post hoc. GFP expressing control mice spent equal amounts of time in both sides of the chamber (51.32 ± 1.97% in the stimulation side, 47.68 ± 1.98% time in the non-stimulation side, *p* = 0.7644). However, ChR2-expressing mice exhibited obvious aversion to the area of the arena programmed for photostimulation of vGlut2^BF→LHb^ circuitry, spending significantly less time in the stimulation side compared to the non-stimulation side (27.84 ± 4.82% in stimulation side, 71.1 ± 4.7% in non-stimulation side, *p* =  < 0.0001; Fig. 3e, f). Moreover, the average visit duration to the stimulation side was significantly shorter for ChR2 mice compared to GFP controls (7.71 ± 1.79 s for ChR2-EYFP mice, 17.5 ± 3.98 s for GFP controls, *p* = 0.0447; Fig. 3g). However, GFP controls and ChR2-EYFP mice had a similar number of visits to the stimulation zone (Fig. 3h), indicating ChR2 mice continued to venture into the stimulation zone despite the elicited aversion. Together, these observations suggest that vGlut2^BF→LHb^ circuitry drives overt real-time place aversion, as ChR2 mice spend overall less time and take shorter visits to the stimulation zone during a real-time place avoidance assay.

### Activation of basal forebrain-to-lateral habenula circuitry impairs memory formation

**Figure 4:**
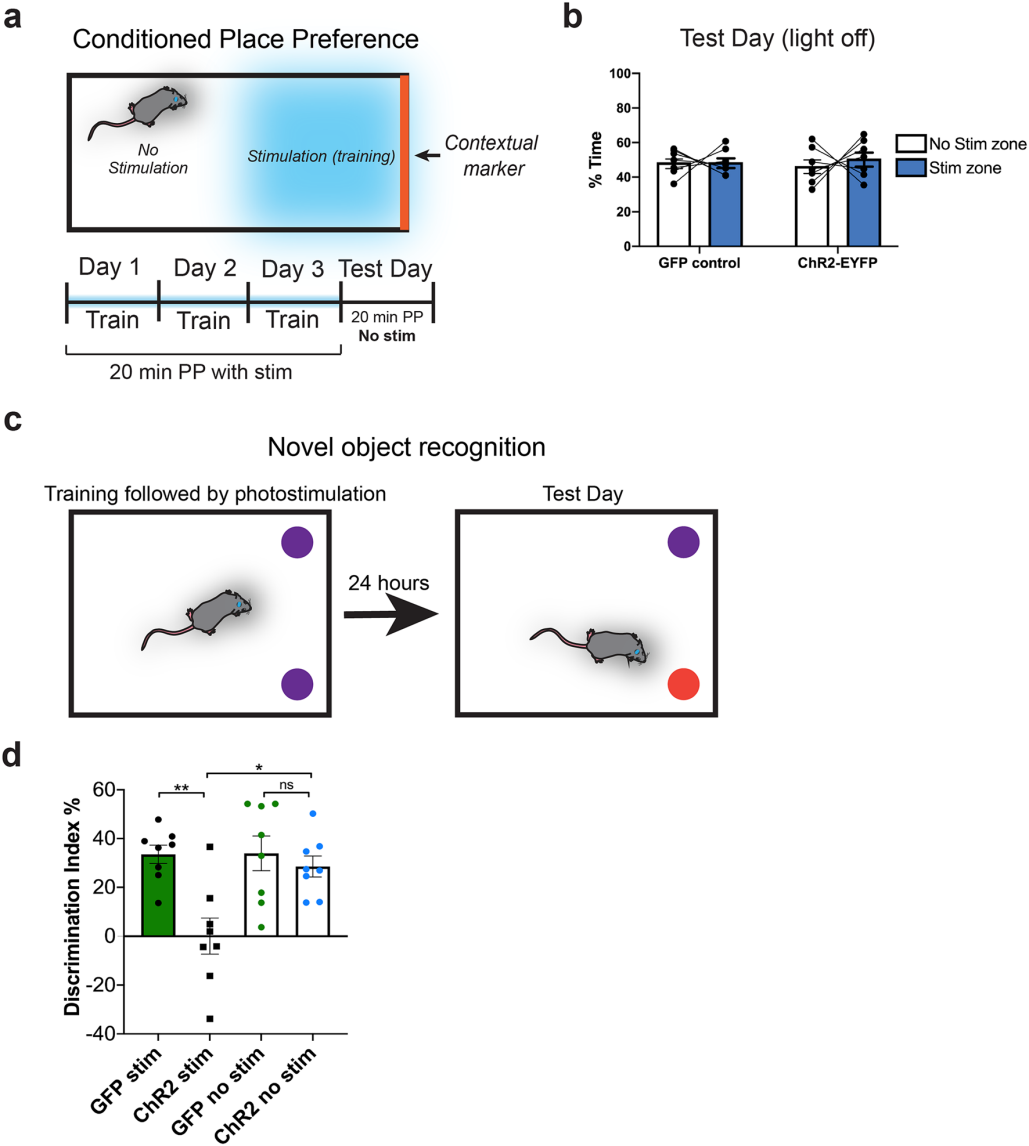
Optogenetic stimulation of basal forebrain-to-lateral habenula circuitry impairs memory formation. (**a**) Contextual conditioned place preference experimental paradigm with optogenetic stimulation. (**b**) Average percent time GFP and ChR2-EYFP optogenetic animals spent in either side of the contextual conditioned place preference arena on test day, without photostimulation. Statistical significance determined using Binomial test for proportion with Bonferroni correction; null hypothesis = 50%. Error bars represent SEM. n = 7. For GFP controls, they spent 48.66 ± 2.798% in non-stim zone, 48.7 ± 2.83% in stim zone, *p* = 0.99. For ChR2 animals, they spent 46.43 ± 3.958% in non-stim side, 50.78 ± 4.07% in stim side, *p* = 0.6137. (**c**) Novel object recognition experimental paradigm. In the photostimulation version of this paradigm, mice were photostimulated (20 Hz, 5 ms pulses) for 10 min after a 10 min training session with two identical objects. One day later, mice were tested on how well they discriminated between one novel and one familiar object. (**d**) Discrimination index (calculated as: [(time investigating novel object – time investigating familiar object)/ total investigation time]*100) on test day of novel object recognition. A discrimination index over 0 indicates a preference for investigating a novel object, as expected. DI for GFP stimulation = 33.53 ± 3.80%. DI for ChR2 stimulation = 0.049 ± 7.38%. DI for GFP no stimulation = 33.91 ± 7.13%, DI for ChR2 no stimulation = 28.55 ± 4.29%. Statistical significance calculated using a repeated measures Two-way ANOVA with a Bonferroni multiple comparisons correction. GFP stim vs ChR2 stim *p* = 0.0023. GFP no stim vs ChR2 no stim *p* =  > 0.9999. ChR2 stim vs ChR2 no stim *p* = 0.0113. GFP stim vs GFP no stim *p* =  > 0.9999.

Due to the dramatic aversion phenotype observed with activation of vGlut2^BF→LHb^ circuitry, we next questioned if this circuitry would lead to learned aversive responses after associative conditioning. To test this, we optogenetically activated vGlut2^BF→LHb^ neurons when animals were in one half of an arena containing a contextual marker (walls with a striped pattern; Fig. 4a), and subsequently measured their ability to associate the cue with photostimulated aversion of vGlut2^BF→LHb^ circuitry. For this, mice were trained for 20 min per day, three days in a row using a conditioned place-aversion paradigm (Fig. 4a). On the test day (day 4), animals were placed in the same chamber as the preceding days but were not subjected to photostimulation. Total activity was recorded for 20 min, and time spent in either side of the chamber was analyzed post hoc. Despite being averse to photostimulation during a real-time place avoidance assay (Fig. 3d–h), mice did not learn to avoid the marked side of an arena on the test day after conditioned place preference training, and spent nearly 50 percent of their time in either half of the arena (Fig. 4b; ChR2-EYFP mice spent 46.43 ± 3.96% of their time in the non-striped side and 50.78 ± 4.07% of their time in the striped/previously stimulated side, *p* = 0.6137). As a positive control to verify that vGlut2^BF→LHb^ optogenetic mice did not have impaired memory formation due to the invasive fiber implants and/or viral injection, we performed contextual fear conditioning using a foot-shock without photostimulation (Supp. Fig. 8a). All mice exhibited a significant increase in the percentage of time freezing in the foot-shock conditioning chamber both 2 and 24 h post-conditioning (Supp. Fig. 8b), indicating vGlut2^BF→LHb^ optogenetic mice have intact learning and memory circuits.

Due to the lack of conditioned place-preference despite potent aversion produced by optogenetic stimulation of vGlut2^BF→LHb^ circuitry, we questioned if this circuitry was bypassing learning and memory of an aversive state and/or location, or if it was potentially disrupting memory formation. To test this, we performed a novel object recognition memory task in which object presentation was followed by optogenetic stimulation (as to not confound the training process). Interestingly, ChR2-expressing mice did not discriminate a “familiar” object from a novel object, with a discrimination index of 0.049 ± 7.38%, indicating that stimulation of vGlut2^BF→LHb^ circuitry occludes memory formation. This effect was reversible, as the same animals adequately discriminated between novel and familiar objects when trained without photostimulation (Fig. 4c, d). Together, these data show that while vGlut2^BF→LHb^ circuitry mediates potent aversion, this aversion is not formed into a memory and cannot be associated with contextual cues like many other types of aversive behaviors. Additionally, stimulation of this circuitry after training actively inhibits memory formation.

### Activation of glutamatergic basal forebrain projections to the lateral habenula does not induce a physiological stress response

**Figure 5:**
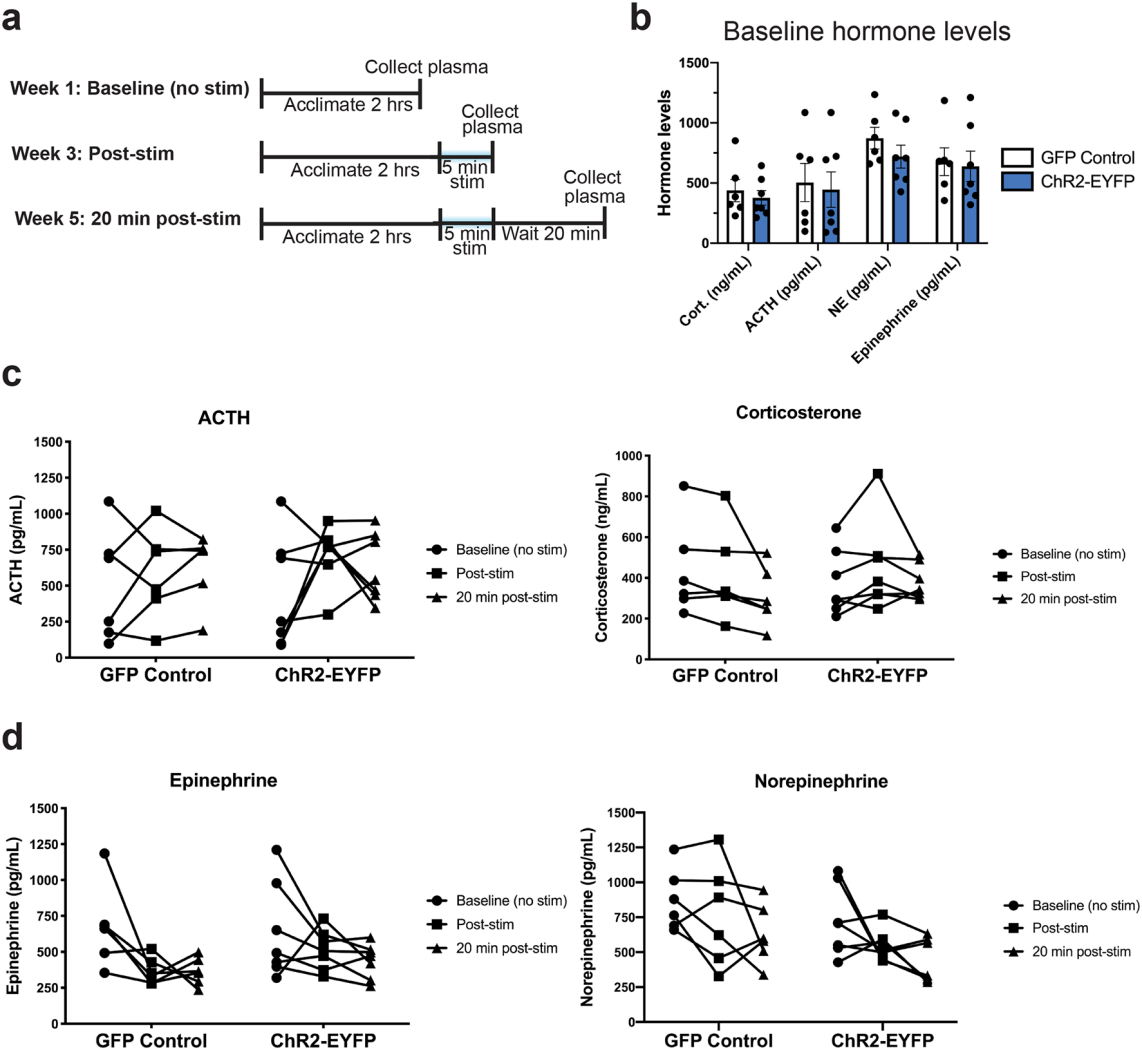
Aversion elicited by basal forebrain-to-lateral habenula activation does not evoke a stress response. (**a**) Experimental paradigm for plasma collection at baseline, post-stim, and 20 min post stim. Each time point separated by 2 weeks. (**b**) Baseline hormone levels across all hormones measured for GFP controls and ChR2-EYFP animals. Statistical tests using multiple unpaired t-tests. Error bars represent SEM. n = 6 for GFP controls, n = 7 for ChR2-EYFP animals. For ACTH: GFP controls = 504.03 ± 158.94 pg/mL, ChR2 animals = 444.82 ± 146.80 pg/mL (*p* = 0.9799). For corticosterone: GFP controls = 437.74 ± 93.38 ng/mL, ChR2 animals = 376.45 ± 60.59 ng/mL (*p* = 0.9111). For epinephrine: GFP controls = 677.17 ± 115.04 pg/mL, ChR2 animals = 639.29 ± 126.13 pg/mL (*p* = 0.9950). For norepinephrine: GFP controls = 873.17 ± 89.83 pg/mL, ChR2 animals = 719.29 ± 94.81 pg/mL (*p* = 0.6007). (**c**) Hormone levels at baseline, post-stim, and 20 min post stim for stress response hormones ACTH and corticosterone. Statistical significance determined using repeated measures Two-way ANOVA with a Sidak correction for multiple comparisons. n = 6 for GFP controls, n = 7 for ChR2-EYFP animals **For ACTH**: GFP controls at baseline = 504.03 ± 158.94 pg/mL, ChR2-EYFP animals at baseline = 444.82 ± 145.80 pg/mL, *p* = 0.9799. GFP controls post stim = 586.11 ± 129.50 pg/mL, ChR2-EYFP post stim = 719.61 ± 77.57 pg/mL, *p* = 0.8197. GFP controls 20-min post stim = 630.24 ± 98.01 pg/mL, ChR2-EYFP 20 min post stim = 628.4 ± 89.46 pg/mL, *p* =  > 0.999. **For corticosterone**: GFP controls at baseline = 437.74 ± 93.38 ng/mL, ChR2-EYFP animals at baseline = 376.45 ± 60.59 ng/mL, *p* = 0.9111. GFP controls post stim = 409.17 ± 92.20 ng/mL, ChR2-EYFP post stim = 455.90 ± 84.11 ng/mL, *p* = 0.9575. GFP controls 20 min post stim = 306.86 ± 58.40 ng/mL, ChR2-EYFP 20 min post stim = 381.12 ± 33.80 pg/mL, *p* = 0.8541. (**d**) Hormone levels at baseline, post-stim, and 20 min post stim for fight-or-flight hormones epinephrine and norepinephrine. Statistical significance determined using repeated measures Two-way ANOVA with a Sidak correction for multiple comparisons. n = 6 for GFP controls, n = 7 for ChR2-EYFP animals. For epinephrine: GFP controls at baseline = 677.17 ± 115.04, ChR2-EYFP animals at baseline = 639.29 ± 126.13, *p* = 0.9950. GFP controls post stim = 365.50 ± 37.82, ChR2-EYFP animals post stim = 514.14 ± 53.00, *p* = 0.1278. GFP controls 20 min post stim = 364.50 ± 39.10, ChR2-EYFP animals 20 min post stim = 438.00 ± 45.31, *p* = 0.5698. For norepinephrine: GFP controls at baseline = 873.17 ± 89.83, ChR2-EYFP animals at baseline = 719.29 ± 94.81, *p* = 0.6007. GFP controls post stim = 768.83 ± 150.05, ChR2-EYFP animals post stim = 546.86 ± 42.95, *p* = 0.4999. GFP controls 20 min post stim = 628.33 ± 87.80, ChR2-EYFP animals 20 min post stim = 431.29 ± 58.80, *p* = 0.2591.

Due to the dramatic aversion phenotype observed with activation of vGlut2^BF→LHb^ neurons, we next asked if activation of this circuitry promoted a stress or fight-or-flight like response typical of other highly aversive states. To test this, we assayed levels of the plasma hormones ACTH, corticosterone, norepinephrine, and epinephrine following optogenetic activation of vGlut2^BF→LHb^ neurons. Blood was collected at baseline (without optogenetic stimulation), immediately following 5 min of photostimulation to detect fast-acting fight-or-flight responses, and 20 min after a 5 min photostimulation period to identify any potential slower acting stress responses. Each time point was separated by 2 weeks to allow for full recovery, and to avoid confounding later time points (Fig. 5a). After isolating plasma from total blood collected, we measured ACTH and corticosterone using radioimunnoassays, and catecholamines via HPLC. Importantly, there were no differences in hormone levels between GFP controls and ChR2-EYFP experimental animals at baseline (without optogenetic stimulation; Fig. 5b). Interestingly, we detected no major differences between GFP controls and ChR2-EYFP mice in any of the hormones measured immediately post-stimulation or 20 min post-stimulation (Fig. 5c, d). In fact, some hormones, such as epinephrine, exhibited a reduction in levels over the course of the experiment, but this effect was observed in both the GFP and ChR2-EYFP groups, potentially indicating an acclimation to the blood-draw procedure (Fig. 5d). Thus, activation of vGlut2^BF→LHb^ neurons elicits robust aversive behavior without inducing a stress or fight-or-flight response. Alongside evidence that this photo-evoked aversive state is not able to be contextualized and impairs memory (Fig. 4), these data suggest that vGlut2^BF→LHb^ circuitry induces an instantaneous, “reflex-like” aversion response.

### Activation of lateral habenula-projecting glutamatergic basal forebrain neurons overrides the drive to eat but does not affect appetite

**Figure 6:**
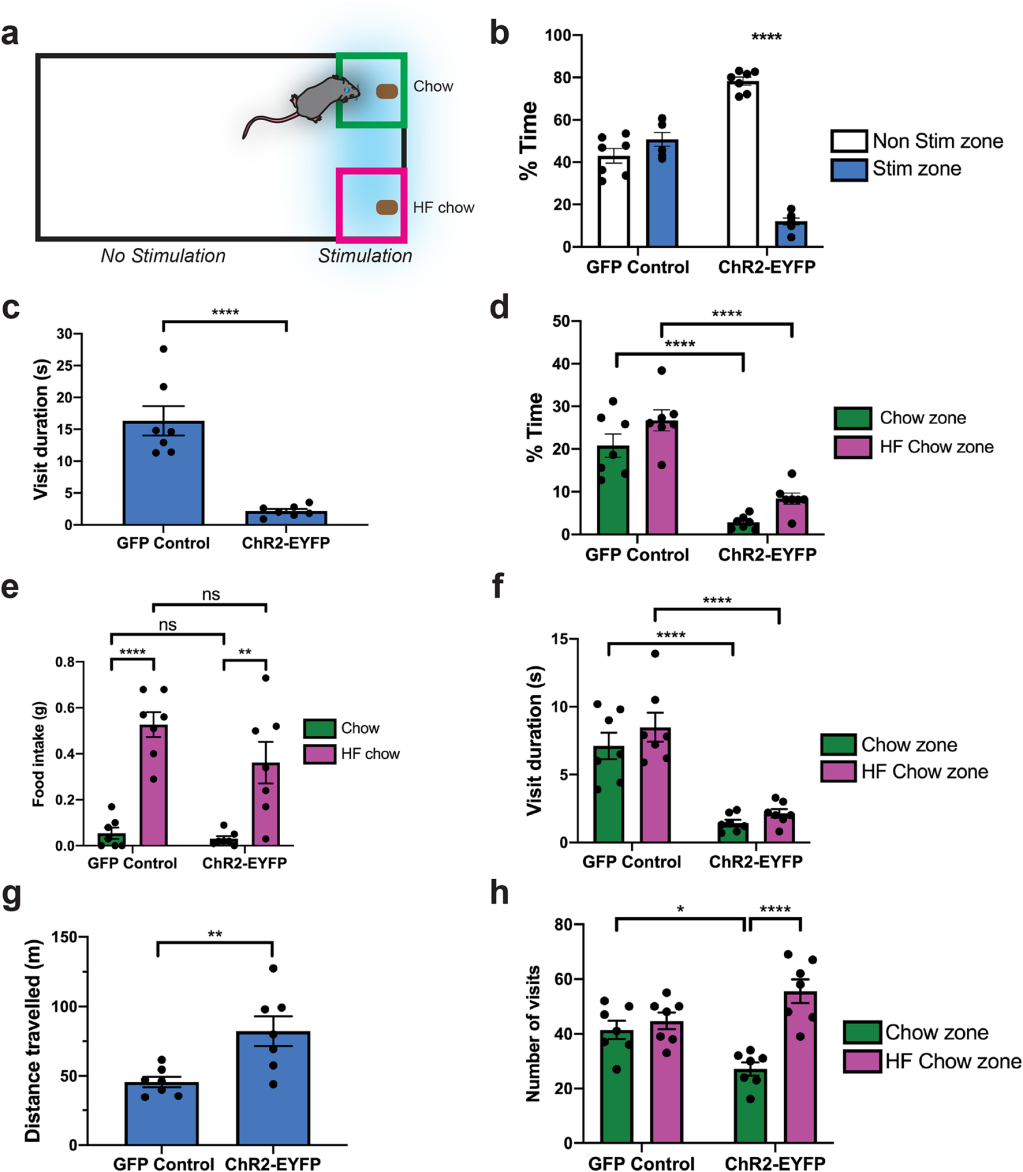
Activation of lateral habenula-projecting glutamatergic basal forebrain neurons overrides the drive to eat, but does not affect appetite. (**a**) Experimental setup for optogenetic stimulation of vGlut2^BF→LHb^ neurons paired with food. (**b**) Average percent time GFP controls and ChR2-EYFP animals spent in either the stimulation or non-stimulation portions of the arena during a 20 min experiment. Error bars represent SEM. Statistical significance determined using Binomial test for proportion with Bonferroni correction; null hypothesis = 50%. n = 7. GFP controls spent 43.00 ± 3.45% time in the non-stimulation side and 50.71 ± 3.22% time in the stimulation/food side (*p* = 0.4705). ChR2-EYFP animals spent 78.29 ± 1.89% time in the non-stimulation side compared to 11.98 ± 1.60% time in the stimulation/food side (*p* =  < 0.0001). (**c**) The average duration of each visit to the stimulation side of the chamber for GFP and ChR2-EYFP animals. Error bars represent SEM. Statistical significance determined using an unpaired, two-tailed t-test. n = 7. GFP animals spent 16.33 ± 2.3 s per visit. ChR2-EYFP animals spent 2.157 ± 0.33 s per visit. *p* =  < 0.0001. (**d**) The percent time GFP controls and ChR2-EYFP animals spent interacting with either chow or high fat chow (within the stimulation portion of the arena). Error bars represent SEM. Statistical significance determined using Two-way ANOVA with a Tukey correction for multiple comparisons. n = 7. In chow zone: GFP controls spent 20.79 ± 2.74% time, ChR2-EYFP animals spent 2.80 ± 0.58% time (*p* =  < 0.0001). In high-fat chow zone: GFP controls spent 26.73 ± 2.45% time, ChR2-EYFP animals spent 8.41 ± 1.3% time (*p* =  < 0.0001). (**e**) Average cumulative food intake of GFP controls and ChR2-EYFP animals throughout the 20 min experiment. Error bars represent SEM. Statistical significance determined using Two-way ANOVA with Tukey correction for multiple comparisons. n = 7. Chow consumed: GFP controls = 0.05 ± 0.03 g, ChR2-EYFP animals = 0.03 ± 0.01 g (*p* = 0.9888). HF chow consumed: GFP controls = 0.53 ± 0.05 g, ChR2-EYFP animals = 0.36 ± 0.09 g (*p* = 0.1640). GFP chow vs. GFP HF chow: *p* =  < 0.0001. ChR2 chow vs. ChR2 HF chow: *p* = 0.0013. (**f**) The average duration of each visit to either the chow or high fat chow zones for GFP controls and ChR2-EYFP animals. Error bars represent SEM. Statistical significance determined using Two-way ANOVA with Tukey correction for multiple comparisons. n = 7. Avg visit to chow zone: GFP controls = 7.11 ± 0.97 s, ChR2-EYFP animals = 1.43 ± 0.25 s (*p* =  < 0.0001). Avg visit to high-fat chow zone: GFP controls = 8.49 ± 1.10 s, ChR2-EYFP animals = 2.14 ± 0.31 s (*p* =  < 0.0001). (**g**) The average distance traveled of GFP controls and ChR2-EYFP animals during the 20 min food choice experiment. Error bars represent SEM. Statistical significance determined using unpaired, two-tailed t-test. n = 7. GFP controls = 45.66 ± 9.94 m, ChR2-EYFP animals = 82.21 ± 28.33 (*p* = 0.0074). (**h**) The average number of visits to either the chow or high fat chow zones of the arena for GFP controls and ChR2-EYFP animals. Error bars represent SEM. Statistical significance determined using Two-way ANOVA with Tukey correction for multiple comparisons. n = 7. Trips to chow zone: GFP controls = 41.43 ± 3.36, ChR2-EYFP = 27.14 ± 2.42 (*p* = 0.0286). Trips to high-fat chow zone: GFP controls = 44.7 ± 3.1, ChR2-EYFP animals = 55.57 ± 4.31 (*p* = 0.1285). Comparing GFP chow vs. GFP HF chow: *p* = 0.8987. Comparing ChR2-EYFP chow vs. ChR2-EYFP HF chow: *p* =  < 0.0001.

Given that activation of vGlut2^BF→LHb^ circuitry induces a potent aversion behavior and overrides hunger-induced feeding, we next asked whether optogenetic activation of this circuitry would be sufficient to prevent fasted mice from consuming high fat (HF; 60% kcal fat) chow, which is highly palatable and rewarding to mice. Prior to the experiment, vGlut2^BF→LHb^ optogenetic mice were habituated to HF chow by supplementing their diet every day for three days. Overnight-fasted mice were then placed in an arena where one end of the arena had two food zones: one with regular chow, and the other with HF chow. Upon crossing into either food zone, fasted mice would receive photostimulation, but did not receive photostimulation in the rest of the arena (Fig. 6a). Mice were video recorded, and food consumption was measured for a total of 20 min. As before, ChR2-expressing mice exhibited a strong aversion to the photostimulation side of the chamber and spent most of their time in the non-stimulation side (Fig. 6b; ChR2 mice spent 78.29 ± 1.89% of their time in the non-stimulation side compared to 11.98 ± 1.60% of their time in the stimulation/food side, *p* =  < 0.0001). ChR2 mice also spent significantly less time per visit to the stimulation side of the chamber (Fig. 6c; ChR2 mice spent 2.16 ± 0.33 s per visit while GFP mice spent 16.33 ± 2.3 s per visit, *p* =  < 0.0001), and thus less time in either the chow or HF chow zones compared to GFP controls. In fact, ChR2 mice spent 2.80 ± 0.58% of their time in the chow zone and 8.41 ± 1.30% of their time in the HF chow zone, while GFP controls spent 20.79 ± 2.74% of their time in the chow zone and 26.73 ± 2.45% of their time in the HF chow zone (Fig. 6d). However, despite the aversion to the photostimulation zone, ChR2 animals consumed the same total amount of either normal or HF chow as GFP controls (Fig. 6e; GFP mice consumed 0.05 ± 0.03 g chow and 0.53 ± 0.05 g HF chow while ChR2 mice consumed 0.03 ± 0.01 g chow and 0.36 ± 0.09 g HF chow). Notably, ChR2 mice made much shorter visits to either the chow or HF chow zones (Fig. 6f), and traveled a further total distance than GFP controls (Fig. 6g). Thus, ChR2 mice adapted their foraging strategy to make shorter trips to food zones, avoiding photostimulation between feeding bouts. ChR2 mice also took more disproportionately more trips to the HF chow zone (Fig. 6h). These data suggest that under the pressure of aversive-linked vGlut2^BF→LHb^ photostimulation, ChR2 animals choose to forage for higher-calorie/more rewarding food. Additionally, ChR2 mice consumed the same amount of food as GFP controls in less time by taking shorter, but more frequent trips to the food zone, and consuming food at a faster rate. While these data partially show the behavioral flexibility of mice to obtain food efficiently when faced with external pressure, they also reveal a fundamental distinction: while the aversion elicited by vGlut2^BF→LHb^ circuitry is sufficient to reduce time spent interacting with food and reduces food intake acutely (Fig. 3), transient activation of this circuitry does not affect appetite. That is, vGlut2^BF→LHb^ circuitry drives aversion that may override feeding behavior when activated, but this activation itself does not reduce appetite, or the motivation to eat.

### Inhibition of lateral habenula-projecting glutamatergic basal forebrain neurons does not affect feeding or reward-related behaviors

While channelrhodopsin reveals whether a circuit is sufficient for a particular behavior, we also tested necessity of vGlut2^BF→LHb^ circuitry for aversion and feeding behavior through optogenetic inhibition, targeting Cre-dependent archaerhodopsin (ArchT-GFP) to the BF and fiber optics over the LHb of vGlut2-Cre^+/−^ animals to inhibit vGlut2^BF→LHb^ terminals. Electrophysiological experiments validated that ArchT-driven photoinhibition of BF terminals effectively inhibits synaptic transmission to LHb target cells (Supp. Fig. 9; see methods). Upon performing in vivo behavioral assays, optogenetic inhibition of vGlut2^BF→LHb^ terminals did not result in a real-time place preference phenotype, nor did it affect food intake in a re-feeding assay (Supp. Fig. 10 and 11). Due to redundancy of feeding and aversion circuits in the brain^27^, it is likely that vGlut2^BF→LHb^ circuitry is sufficient, but not singularly necessary, to regulate proper feeding and aversive behaviors. Nonetheless, our data clearly identify an aversion circuit capable of overriding the drive eat in a rapid and reflexive way.

## Discussion

**Figure 7:**
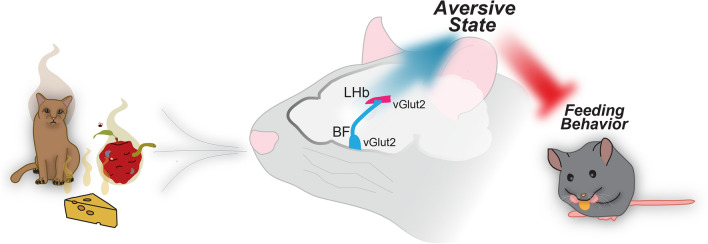
Graphical abstract. Olfactory sensory information is relayed to the LHb via BF glutamatergic circuitry to drive aversive behaviors, overriding appetitive behaviors such as feeding.

In this study we have identified that vGlut2^BF^ neurons robustly project to the LHb, a prominent aversion center of the brain. Using fiber photometry and calcium imaging in vivo, we found that vGlut2^BF→LHb^ circuitry responds to diverse sensory information, including aversive and food-related sensory cues. Using optogenetics, we observed that vGlut2^BF→LHb^ projections drive robust aversion, and override food consumption without affecting appetite. This aversion was not remembered after conditioning, nor did it trigger a stress or fight-or-flight response. Activation of vGlut2^BF→LHb^ circuitry also impaired memory formation. Therefore, this BF-to LHb aversion circuitry acts instantaneously in a reflex-like manner (Fig. 7).

The basal forebrain is a node with various functions, including sensory processing, attention, motivation, learning, and memory^18,25,28–35^. Previously, we and others have independently revealed the BF to have roles in appetite suppression and aversive behaviors^12–14,26^. One of the ways the BF may drive distinct behaviors is through its differential projections. Targets of the BF include sensory regions such as the piriform cortex, feeding-associated regions such as the hypothalamus, and reward/aversion-associated regions such as the basolateral amygdala, ventral tegmental area, and lateral habenula^12,13,26^. While this study focused on the role of BF projections to the LHb in aversion and feeding, previous studies have shown that BF projections to the LHA also drive aversion and hypophagia^12^. The LHA is a particularly interesting target of the BF because vGlut2^LHA^ neurons are known to drive aversion and appetite suppression independently as well^10^. However, terminal-field activation of vGlut2^BF→LHA^ projections does not fully recapitulate the aversion induced by BF cell bodies. This indicates that other downstream nodes—such as the LHb—may work in concert with the LHA to modulate such behaviors.

The LHb is known to be involved in aversion and escape behaviors, is activated by stress and punishment, as well as punishment-predicting sensory cues, and overall drives an aversive state through its inhibition of both the motivation and reward driven mesolimbic dopaminergic and dorsal raphe serotonergic systems^15,36–42^. With regards to feeding, LHA projections to the LHb inhibit hedonic feeding and drive aversive behaviors^9^. Thus, the LHb serves as a prime candidate for the convergence of aversion and feeding circuitry. We found that activating vGlut2^BF^ projections to the LHb suppresses the drive to eat and elicits real-time place aversion. Numerous other inputs to the lateral habenula result in aversion when stimulated, including the lateral preoptic area, ventral pallidum, medial septum, and the lateral hypothalamus^9,43–52^. There have also been reports of reciprocal projections from the LHb to the LHA^36^. Reciprocal connectivity between these two nodes may synchronize different aversion centers in the brain to ensure a swift, rapid behavioral response to threatening or maladaptive stimuli. With the BF being upstream and functionally connected to both the LHb and LHA, the BF could provide further synergistic control of aversion and facilitate this synchronization. Thus, the interconnectivity of the BF, LHA, and LHb provides an interesting tri-node circuit by which aversive and appetitive information is synchronized to drive rapid changes in behavioral state. It is important to note that most of the manipulations in this study involved gain-of-function optogenetic experiments using channelrhodopsin to activate BF axon terminals. One potential caveat to this method of experimentation is the possibility of back-propagating action potentials. However, the hypophagic phenotypes of BF optogenetic stimulation observed previously^12^ differ from the broader aversion phenotypes observed in this study, indicating this is likely not the case in our experiments. Also, the inability to promote feeding and/or suppress aversion via terminal field inhibition within the LHb via targeted archaerhodopsin stimulation does not definitively demonstrate a bona fide loss-of-function manipulation. This may be that this node of circuitry alone is not able to control such behaviors, or it also may be considered a technical hurdle to effectively silence communication at this node using this approach.

The BF plays important roles in sensory processing and integration, and has been shown to respond to aversive and appetitive sensory cues^12,17,18,30–32,34^. Using genetically encoded calcium indicators and fiber photometry, we observed that vGlut2^BF→LHb^ projections, as well as LHb cells receiving BF input, respond to diverse sensory stimuli. The LHb has been previously shown to be activated by various types of sensory stimuli, including aversive stimuli and aversive predictive cues, as well as rewarding cues^41,44,53–55^. Thus, one route that relays diverse sensory information to the LHb is likely via the BF.

Diverse sensory stimuli activate both vGlut2^BF→LHb^ axon terminals and LHb cells that receive BF input. Of note, it is interesting that LHb cellular responses were much sharper and greater in magnitude than BF terminal responses. This may be a technical aspect due to differences in signal-to-noise when recording from soma versus axon terminals, or it may indicate physiological differences. For example, BF terminals may be less synchronized in their responses to sensory cues, elongating the odor response, while LHb cells may respond more uniformly and quickly. One critical question is whether it’s the same, or different BF/LHb populations that respond to aversive and appetitive sensory cues, respectively. By measuring only summed population responses via fiber photometry, it is difficult to address this question. Both nodes are composed of heterogeneous cell types, and different subpopulations may respond to rewarding and aversive stimuli^53,56^. However, considering these nodes also respond to neutral odors, it is also possible that this circuit responds broadly, or non-selectively, to sensory information. It is possible that vGlut2^BF→LHb^ circuitry is activated by both food-related and aversive odors because it modulates aversion and food intake suppression independently, through distinct mechanisms. Likewise, the response to neutral odors may indicate a role for BF projections in relaying any salient sensory information to reward and aversion centers. Therefore, it will be critical to determine whether vGlut2^BF→LHb^ projections contain functionally distinct ensembles that respond to appetitive or aversive cues, respectively. Or, if vGlut2^BF→LHb^ projections broadly respond to sensory information, it will be critical to determine what other brain regions assign valence to this sensory information—for example, through additional downstream targets of the BF, or through other inputs to the LHb.

Aversive stimuli normally form associations with different sensory and contextual cues. Additionally, such drastic aversive behaviors would be expected to drive a stress response. The inability for vGlut2^BF→LHb^ aversion to be associated with contextual cues or generate a hormonal stress response is therefore somewhat surprising. However, independent studies have revealed aversion elicited by BF cell bodies also does not drive conditioned place preference^26^, indicating that BF-driven aversion does not appear to be remembered. Even more surprising, optogenetic stimulation of vGlut2^BF→LHb^ circuitry following novel object recognition training completely impaired object discrimination, indicating that vGlut2^BF→LHb^ circuitry actively inhibits memory formation. While provocative, this raises the question of what the evolutionary purpose of a circuit that drives instantaneous aversion and impairs memory consolidation would be. Perhaps the reflexive, instantaneous nature of vGlut2^BF→LHb^ driven aversion stems from this circuit’s ability to disrupt all behavioral and cognitive function, facilitating an immediate aversive response. Once this signaling is absent, cognitive functions such as learning and memory, or motivated behaviors such as feeding, may ensue. Other studies have also indicated that either activation or inhibition of the LHb impairs contextual conditioning^57^, so perhaps any disturbance to LHb signaling is sufficient to impair learning and memory. These effects on memory align with our observations that ChR2-expressing mice continuously return to a photostimulation zone during real-time place avoidance assays, making the same number of trips as GFP controls despite the elicited aversion. One possible interpretation of these data is that activating vGlut2^BF→LHb^ circuitry increases impulsivity, which makes mice unable to learn, and causes them to repeatedly enter an aversive zone. Finally, since ChR2-expressing mice resume normal food consumption immediately following optogenetic stimulation, activating vGlut2^BF→LHb^ circuitry does not appear to stress the animal, as acutely stressed animals lose their appetite^58^. Taken together, the learning and memory assays and hormone data indicate vGlut2^BF→LHb^ circuitry drives reflexive aversion that is distinct from other aversive behaviors.

When challenged to consume food in an area of aversive photostimulation, vGlut2^BF→LHb^ optogenetic mice adapt their behavioral strategy to make short, but frequent trips to a food zone so they can consume as much food as possible in a short time, resulting in similar food intake levels as controls. Since cumulative food intake is not affected, this indicates that activating vGlut2^BF→LHb^ circuitry inhibits food intake without affecting appetite or overall food intake. This result contrasts with the obvious feeding suppression when mice are stimulated continuously throughout an arena (as in Fig. 3c). One explanation for these contrasting results is that when mice are only stimulated in a specific zone of an arena, they are able to initiate feeding behavior in short bouts, escaping to a non-stimulation zone once the aversive photostimulation is maximally activated. On the other hand, when photostimulated continuously, mice never initiate feeding and therefore consume less food compared to GFP controls. These data support the interpretation that vGlut2^BF→LHb^ circuitry primarily drives an aversive phenotype that overrides feeding behavior without affecting appetite. The suppression of food intake, therefore, is secondary to aversive behavior. However, an alternative interpretation is that vGlut2^BF→LHb^ circuitry has separate functions in both feeding suppression and aversion. This alternative interpretation is supported by fiber photometry data that reveal odor responses to both food-related and aversive cues, as well as data showing a lack of a hormonal stress response after optogenetic stimulation, as many aversion circuits do trigger fight-or-flight and stress hormone release. Perhaps feeding suppression and aversion are normally parsed apart via different firing rates, magnitude of neuronal response, or recruitment of different neuronal ensembles. However, the artificial nature of optogenetic stimulation, in which the entire BF→LHb population fires at a specific frequency, may mask physiological vGlut2^BF→LHb^ signaling. Nonetheless, these data indicate vGlut2^BF→LHb^ circuitry is sufficient to drive both potent, reflexive aversion and drastic feeding suppression.

In sum, we have identified and interrogated a basal forebrain circuit that drives potent, reflexive aversion that overrides feeding behaviors. The reflexive nature of this aversion is unique, and alters our understanding of how aversion circuits compete with motivated behaviors to drive rapid responses to innately aversive stimuli. Additionally, this study provides critical insight as to how aversion/reward and sensory processing interacts with homeostatic circuits to alter feeding behavior and body weight. From an evolutionary perspective, motivated behaviors essential for survival, such as foraging, feeding, and reproduction, must be balanced by caution of novel and/or foreign environments, and an awareness of environmental cues that would indicate reward or danger. Thus, numerous aversion and escape neural circuits in the brain must be wired to override feeding circuits when necessary if a threat is present. By studying how these circuits function and are anatomically connected, we can better understand and treat devastating disorders such as obesity and eating disorders, which have major direct effects on health, as well as numerous other detrimental side effects^59,60^. Additionally, due to the LHb’s involvement in addiction, mood disorders, and schizophrenia^15,61^, understanding the influence of BF circuitry on this aversion node can potentially aid in our understanding of other types of mental illnesses.

## Materials and methods

Mice:

- vGlut2-Cre (Slc17a6^tm2(cre)Lowl^)
  - Source: Jackson laboratory
  - Strain #: 016963
- Wildtype C57BL/6NJ
  - Source: Jackson laboratory
  - Strain #: 005304

AAVs:

- Cre-dependent synaptophysin: AAV-Ef1α-flex-Synaptophysin::mRuby2-WPRE-hGHpA
  - Serotype: DJ8
  - Source: Neuroconnectivity Core at the Jan and Dan Duncan Neurological Research Institute
- Cre-dependent ChR2: AAV-Ef1α-flex-hChR2(H134R)-EYFP-WPRE-hGHpA
  - Serotype: 2/9
  - Source: Neuroconnectivity Core at the Jan and Dan Duncan Neurological Research Institute; plasmid subcloned from Addgene #26973
- Cre-dependent mRuby: AAV-Ef1α-flex-mRuby2
  - Serotype: DJ8
  - Source: Neuroconnectivity Core at the Jan and Dan Duncan Neurological Research Institute; plasmid subcloned from Addgene #40260
- Cre-dependent GFP: AAV-Ef1α-flex-eGFP
  - Serotype: DJ8
  - Source: Neuroconnectivity Core at the Jan and Dan Duncan Neurological Research Institute; plasmid subcloned from Addgene #28304
- Cre-dependent GcaMP: AAV-Syn-flex-GcaMP8s-WPRE-hGHpA
  - Serotype: DJ8.
  - Source: Neuroconnectivity Core at the Jan and Dan Duncan Neurological Research Institute; plasmid from Addgene #100839^62^
- AAV1-Cre: AAV1-hSyn-Cre-WPRE-pA
  - Serotype: 2/1 (anterograde)
  - Source: Addgene #105553 (U Penn Viral Core)
- Cre-dependent ArchT: AAV-CAG-flex-ArchT-GFP
  - Serotype: 2/9
  - Source: Neuroconnectivity Core at the Jan and Dan Duncan Neurological Research Institute; plasmid from Adddgene #28307 from Edward Boyden’s laboratory^63^

### Animals

Mice used in this study were treated in compliance with US Department of Health and Human services, and IACUC. All experimental protocols were approved by Baylor College of Medicine and the licensed BCM IACUC protocol approval committee under protocol number AN5596. All methods have been reported in accordance with recommendations in the ARRIVE guidelines. For all experiments, both male and female littermates were used, and were distributed across both experimental and control groups. Mice were at least 8 weeks old for stereotaxic surgeries, and behavior was performed on mice between 3 and 5 months of age. Animals were maintained on a 12 h light/dark cycle and were group housed. Mice were fed standard mouse chow (Harlan, 2920X), and this chow or high fat chow (60% kcal fat, Research Diets Inc 12492) was used for feeding experiments. vGlut2-Cre (Slc17a6^tm^^2^^(cre)Lowl^/J Stock No. 016963^64^) mice were purchased from Jackson Laboratories. Genotyping for vGlut2-Cre was done using the following primers from Jackson Laboratories: Mutant Reverse “ACA CCG GCC TTA TTC CAA G” (Primer 13007), Common “AAG AAG GTG CGC AAG ACG” (Primer 32667), and Wild type Reverse “CTG CCA CAG ATT GCA CTT GA” (Primer 32668). To obtain heterozygotes (vGlut2-Cre^+/−^), vGlut2-Cre homozygous mice (vGlut2-Cre^+/+^) were crossed to C57BL/6NJ wildtype mice (Jackson labs Stock No. 005304).

### Stereotaxic viral injections

For all stereotaxic surgeries mice were anesthetized and maintained under anesthesia using ~ 1–3% vaporized isoflurane with oxygen. A stereotaxic instrument connected to Angle Two software was used to accurately target regions of the brain. After leveling the skull both in the ML and AP directions (within + /− 0.03 mm), different regions were targeted using empirically determined coordinates. The AP coordinate was shifted slightly anterior to adjust for the tilt of the brain, and optimized coordinates were verified using diI injections prior to any stereotaxic surgery experiment. Towards this, the basal forebrain was targeted through bilateral injections from bregma, AP = 1.18 mm, DV = − 5.8 mm, and ML =  ± 1.29 mm. For all experiments 150 nL of virus was used per side and the virus was concentrated in the medial basal forebrain regions from Bregma 0.97 through Bregma 0.50. For anatomical tracing experiments, AAV-Ef1α-flex-Synaptophysin::mRuby2-WPRE-hGHpA (serotype DJ8) was used. For channelrhodopsin-assisted circuit mapping, AAV-Ef1α-flex-hChR2(H134R)-EYFP-WPRE-hGHpA (serotype 2/9) was used for light-assisted activation, and AAV-Ef1α-flex-mRuby2 (serotype DJ8) was used to label cells to record from. For electrophysiological validation of ArchT-GFP, AAV-CAG-flex-ArchT-GFP (serotype 2/9) was mixed with AAV-Ef1α-flex-hChR2(H134R)-EYFP-WPRE-hGHpA (serotype 2/9) as a 1:1 ratio by volume. For optogenetic behavior assays AAV-Ef1α-flex-hChR2(H134R)-EYFP-WPRE-hGHpA (serotype 2/9) was used for gain-of-function experimental animals, AAV-Ef1α-flex-eGFP (serotype DJ8) was used for controls, and AAV-CAG-flex-ArchT-GFP (serotype 2/9) was used for loss-of-function experiments. For calcium imaging experiments AAV-Synapsin-flex-jGCaMP8s-WPRE-pA (serotype DJ8) and/or AAV1-hSyn-Cre-WPRE-pA (serotype 2/1^23^) was used. All viruses were titered to at least 10^11^ viral particles/µL.

### Immunohistochemistry and microscopy

At a minimum of two-weeks following viral injection, mice were anesthetized using isoflurane and were transcardially perfused with PBS followed by 4% PFA (diluted using 16% paraformaldehyde EM Grade No. 15710 Electron Microscopy Sciences). Brains were dissected out and drop-fixed further in 4% PFA overnight at 4 °C, followed by cryoprotection overnight in 20% sucrose in PBS, and finally overnight in 30% sucrose in PBS at 4 °C. Cryoprotected brains were then embedded and frozen in O.C.T. (Fisher HealthCare No. 4585) and stored at − 80 °C until sectioning. Brains were sliced coronally in the anterior to posterior direction on a cryostat (Leica CM1860) at 40 μm for viral tracing/labeling experiments and 80 μm for determining fiber optic implant sites post hoc. If slicing at 40 μm, every third section was collected. If slicing at 80 μm, every section was collected. After washing with PBS, slices were mounted onto slides and stained using DAPI Fluoromount-G (Southern Biotech, 0100–20). Images were taken using either a Leica TCS SPE confocal microscope at 10 or 20×, a Leica TCS SP8 STED microscope, or a Leica SP8X microscope. All tiled images were taken at 10x. For quantification of BF synaptophysin::mRuby2 labeling, three animals were analyzed across the entire brain, from the olfactory bulb to the cerebellum. In regions in which synaptophysin::mRuby2 labeling was observed, three images were taken per region (identified using the Allen Brain Atlas) across different slices within an animal. The fluorescent signal was quantified post hoc using Imaris, in which masks were drawn over regions of interest (ROIs) and the background signal was subtracted to calculate a volume of synaptophysin::mRuby2 terminals divided by the total volume of the ROI. Technical replicates within an animal were then averaged to create a biological replicate average, which was used for calculating an overall average synaptophysin::mRuby2 density across biological replicates.

### Channelrhodopsin-assisted electrophysiology

Slice electrophysiological recording experiments were performed as previously described^65^ with minor modifications. Briefly, mice were deeply anesthetized with isoflurane and then transcardially perfused with ice-cold artificial cerebrospinal fluid (aCSF) solution containing (in mM): 125 NaCl, 2.5 KCl, 1.25 NaH2PO4, 1 MgCl2, 2 CaCl2, 25 glucose, and 25 bicarbonate (pH 7.3, 295 mOsM). Brains were removed and transferred into ice-cold cutting solution containing (in mM): 2.5 KCl, 1.25 NaH2PO4, 10 MgSO4, 0.5 CaCl2, 234 sucrose, 11 glucose, and 26 bicarbonate. Cutting solution was continuously bubbled with 95% CO2/5% O2. Brains were embedded coronally in 1.5% low melting point agarose. Agar-embedded brains were immediately submerged in oxygenated cutting solution on a Leica VT1200 vibratome. Three-hundred micrometers coronal sections were made at a cutting speed of 0.4 mm/s. Slices were removed to a slice recovery chamber of oxygenated aCSF at 37 °C for at least 30 min. Following recovery, slices were slowly returned to room temperature for 30 min before recording.

For optogenetic circuit mapping of basal forebrain glutamatergic inputs to the LHb, vGlut2+ LHb cells were identified through mRuby2 labeling and were patched. Patched cells were first voltage-clamped at − 65 mV to record baseline membrane properties. To check for the presence of a light-evoked inward current, channelrhodopsin was activated by full-field illumination from a filtered xenon light source filtered to (Olympus, U-N41020). The onset and duration of light stimulation was controlled through ClampEx software (version 10.3) by a mechanical shutter (Sutter). Patched cells were then voltage-clamped at 0 mV (adjusted for junction potential) to reveal outward currents. If a light-evoked outward current was observed in aCSF, then TTX (1 µM), 4AP (0.5 µM), and CNQX (10 µM /APV (50 µM) were serially bath-applied to verify: (1) the action potential-dependence; (2) the monosynaptic nature; and (3) the glutamate receptor-dependence of the evoked current.

To validate our ability to inhibit vGlut2^BF→LHb^ circuitry via ArchT stimulation, we injected a 1:1 mixture by volume of AAV-CAG-flex-ArchT-GFP and AAV-Ef1α-flex-hChR2(H134R)-EYFP-WPRE-hGHpA to the BF, and whole-cell voltage-clamp recordings were performed from cells in the LHb according to the specifications above. Upon identifying those that responded to presynaptic stimulation of ChR2 in BF terminals using 470 nm light, we then tested whether ArchT inhibition suppressed ChR2-evoked firing. Towards this, 565 nm light was continuously delivered over a sustained period. Then, during ArchT photoinhibition, ChR2 was sequentially stimulated (470 nm light) to test whether ArchT suppressed ChR2-evoked firing. Finally, ArchT stimulation was ceased, and ChR2 stimulation was used to reversibly re-activate the postsynaptic LHb cell.

### Fiber optic implantation for calcium imaging

For BF axon terminal calcium imaging experiments, male and female vGlut2-Cre^+/−^ littermates were bilaterally injected with rAAV-Syn-flex-GCaMP8s-WPRE-hGHpA^62^ into the HDB using coordinates described above. For LHb cell body calcium imaging, rAAV1-hSyn-Cre-WPRE-pA was bilaterally injected into the HDB and rAAV-Syn-flex-GcaMP8s-WPRE-hGHpA injected into the left LHb. At the same time as viral injection, a fiber optic implant (200um core with NA = 0.50, RWD R-FOC-L200C-50NA) was secured over the left LHb using coordinates from bregma AP = − 1.45, ML = − 0.45, and DV = − 2.60 using the same process as above. Additionally, an aluminum headplate was cemented to the posterior portion of the skull that would permit head fixation of mice on a running wheel during odor presentation. Mice were allowed to heal and express virus for 3 weeks before fiber photometry recordings ensued.

### Fiber photometry recording during odor presentation

During fiber photometry recordings, mice were head-fixed onto a running wheel with an olfactometer^22^ positioned roughly 6 cm from their nose. Odorants presented included, fox urine (Predator Pee), chow (5V5 feed crushed and dissolved in mineral oil), Methylbutylamine (Sigma 241407), Cadaverine (Sigma 52063), Butyric Acid (Sigma B103500), R(+)-Limonene (Sigma 183164), S(-)-Limonene (Sigma 218367), Rose oil (Rainbow Abby), and Peanut Butter (Justin’s). All odors were dissolved in mineral at 2% concentration by volume and presented in replicates of 10, in a randomized order. Mineral oil alone was used as a negative control. All odors were novel except for the 5V5 chow odorant.

Fiber photometry recordings were done using the Doric system as described in^66^. Briefly, two light emitting diodes (465 and 405 nm wavelength) were coupled to a filter cube by fiber optic cables (400 um core, NA = 0.48). The filter cube separated excitation and emission wavelengths, directing the excitation wavelengths along another fiber optic (200 um core, NA = 0.48) that was connected to the implanted fiber optic on the mouse using a ferrule sleeve. Emission wavelengths were carried from the mouse to the filter cube along the same fiber, then directed to a femtowatt photodetector (Newport) through fiber optic cable (600 um core, NA = 0.48). Excitation and emission were controlled and recorded, respectively, in Doric Studio software. jGCaMP8s was excited at 465 nm to record calcium dynamics indicative of neural activity. Simultaneously exciting at 405 nm, the isosbestic point for GCaMP, we collected emissions that were insensitive to calcium binding. This was used to control for motion artifacts and other calcium-independent noise. To record from the control channel (405 nm) and the experimental channel (465 nm) simultaneously, we employed a “locked-in” strategy where each LED was modulated at a different high frequency (typically 270 and 500 Hz, respectively). Emission resulting from both modes of excitation was recorded by the same photodetector and the signal was demodulated online in Doric Studio to separate the control channel form the experimental channel. Both signals were then converted to dF/F in Doric studio using their analysis tool, subtracting the control channel the experimental channel to reduce noise. Z-scored dF/F was calculated for the 5 s prior and 20 s after each odor presentation. Odors were presented for 2 s followed by an 18 s intertrial interval. Heat maps were generated in MATLAB (version R2019a). Arduino-generated TTL pulses triggered the olfactometer and were used as a digital input during recording to precisely align fiber photometry recording with odor presentation. Once 10 technical replicates were averaged for each individual mouse, biological replicates were averaged together to create a composite average. The average z-score response was calculated for 3 s prior (baseline) and 3 s post-odor delivery. Then, average baseline responses were subtracted from the odor response to generate a baseline-normalized odor response across all odors. Statistical significance was then calculated using a one sample t-test, comparing normalized odor responses to 0 (the null hypothesis).

### Optogenetic stereotaxic fiber optic implantation

For optogenetic implants, eight week old vGlut2-Cre^+/−^ mice were stereotaxically injected bilaterally into the BF with rAAV-Ef1α-flex-hChR2(H134R)-EYFP-WPRE-hGHpA (serotype 2/9), rAAV-Ef1α-flex-GFP (serotype DJ8), or rAAV-CAG-flex-ArchT-GFP (serotype 2/9). One week later, mice were bilaterally implanted over the LHb with custom-made fiber optic implants, as described in Patel and Swanson 2019^67^. Briefly, 200 um core fiber optic cable (0.22 NA, FG200AEA) was stripped and secured into a 230 um ferrule (Thor Labs CFLC2301-10) using UV light-cured epoxy (Bondic). The implant was trimmed to 3.5 mm, and the flat end of the implant polished to ensure high light ouput (at least 1 mW). The lateral habenula was implanted bilaterally at a 15 degree angle at coordinates from bregma AP = − 1.58, ML =  ± 0.34, and DV = − 2.85. Fiber optic implants were secured using cement (C and B Metabond Dental cement, Parkell), and capped with crosslinked flash acrylic (Yates-Motloid 44115 and 44119). 18-gauge needles were trimmed to about 1 inch in length and secured with acrylic over the posterior portion of the skull to attach mice to patch cables without scruffing, and instead using ring forceps to attach mice to patch cables. Mice were allowed to recover for two weeks following implant surgery before behavior experiments.

### Optogenetic feeding behavior

Male and female vGlut2-Cre^+/−^ littermates were stereotaxically injected with rAAV-flex-ChR2-EYFP or rAAV-flex-GFP into the BF and implanted with fiber optics over the LHb to stimulate BF terminals. Following 2 weeks of recovery, mice were fasted overnight. The following day mice were attached to patch cables and allowed to acclimate to a behavior chamber alone for 5 min before beginning the experiment. After acclimation, individual mice were presented with chow and photostimulated with a 473 nm laser (Doric; output at least 1 mW) at 20 Hz with 5 ms pulses for 5 min. After 5 min, the laser was turned off and the food was weighed. Then mice were allowed to consume food without photostimulation for 5 min. This was repeated once more (5 min with photostimulation, followed by 5 min without photostimulation) for a total duration of 20 min, with food intake being weighed every 5 min. Average food intake for each time point was calculated, and ChR2 and GFP control groups were compared via a repeated measures two-way ANOVA.

For ArchT-GFP mice, the same stereotaxic surgery was performed as above but using rAAV-CAG-flex-ArchT-GFP. After recovery, mice were fasted overnight, attached to fiber optic cables, provided with food, and allowed to consume food for 20 min without photostimulation to calculate a baseline food intake (food was weighed every 5 min). The following week, the same mice were fasted overnight, and then photo-inhibited with a 561 nm laser (CrystaLaser) at 1 Hz with 900 ms pulses for 20 min continuously while provided with food. Food was weighed every 5 min. The average food intake for each time point was calculated, and “no stimulation” and “stimulation” groups were compared via a repeated measures two-way ANOVA.

### Real-time and conditioned place avoidance assays

Male and female vGlut2-Cre^+/−^ littermates were stereotaxically injected with rAAV-flex-ChR2-EYFP, rAAV-flex-GFP, or rAAV-CAG-flex-ArchT-GFP into the BF and implanted with fiber optics over the LHb to stimulate BF terminals. Following 2 weeks of recovery, mice were attached to patch cables and placed in a large rectangular arena (25in x 17in). Mice were allowed to acclimate for 5 min, and then the assay began. Animals’ movements were tracked using a camera that interfaced with the open-source software Bonsai^68^, which was able to detect when a mouse was in a pre-determined ROI. This software then triggered a TTL pulse for laser photostimulation (473 nm light, 20 Hz, 5 ms pulses for ChR2 mice or 561 nm light, 1 Hz, 900 ms pulses for ArchT mice) when the mouse was in this region. Using this software, mice were photostimulated on one half of the arena while freely roaming for a total of 20 min, while being video recorded. For conditioned place preference, this paradigm was repeated for 3 days in a row, and on testing day was repeated without photostimulation. For both real-time and conditioned place avoidance, the time spent in either half of the arena was calculated *post-hoc* using the open source software Optimouse in MATLAB^69^. Statistical significance was determined using a Binomial test with the null hypothesis being that mice would spend 50% of their time in both halves of the arena. Heat maps were generated using Noldus EthoVision (XT 16) software.

### Contextual fear conditioning

Contextual fear conditioning was performed as previously described^70^ with some minor modifications. Briefly, mice were handled 3 min per day for 3 days and then habituated to the conditioning chamber for 20 min for two consecutive days. On the training day, mice were placed in a chamber with visual contextual markers on the walls and acclimated for 2 min (naïve). Mice then received 2 foot shocks 90 s apart (0.75 mA, 2 s each). One minute later, mice were returned to their home cages. Two and 24 h later, mice were placed back in the conditioning chamber for 5 min to test short-term and long-term memory, respectively. During this time, freezing response (immobility) was recorded using real-time video analyzed by FreezeView. Statistical significance was measured using a repeated-measures Two-way ANOVA with a Sidak correction for multiple comparisons.

### Novel object recognition

Novel object recognition was performed as was previously described^71^ with minor modifications. Mice were habituated to a black plastic chamber (37 × 37 × 37 cm) and to optogenetic fiber optic cables for 10 min per day for three days prior to training. On training day, mice were attached to fiber optic cables and were allowed to explore two identical objects for 10 min. Following this training, the objects were removed and mice were photostimulated at 20 Hz (5 ms pulses) for 10 min, and then returned to their home cage. 24 h later (the test day), mice were attached to fiber optic cables, and presented with one object from the previous day (the familiar object), and one novel object of roughly the same size (novel object) for 10 min. Using AnyMaze software (version 6.2), the amount of time investigating either object was recorded by trained experimenters blinded to experimental treatment. Mice were defined to be investigating an object if their nose was sniffing within a 2 cm radius of the object. From these data, a discrimination index was calculated by subtracting the time spent investigating the familiar object from the novel object and dividing by the total investigation time as a percentage [(time investigating novel object − time investigating familiar object)/(time investigating novel object + familiar object)] × 100. To test the reversibility of the photostimulation effect, the same assay was performed one week later without photostimulation and with a different set of familiar and novel objects. Statistical significance was determined using a repeated measures Two-way ANOVA with a Bonferroni correction for multiple comparisons.

### Hormone assays

Plasma for hormone measurements was collected at three time points: (1) baseline, in which animals were attached to fiber optic cables and allowed to acclimate for 2 h to wash out handling stress, then removed from cables and blood was immediately collected. (2) post-stimulation, in which animals were attached for fiber optic cables, acclimated for 2 h, stimulated for 5 min at 20 Hz (5 ms pulses), removed and blood immediately collected, or (3) 20 min post-stimulation, in which animals were attached to cables, acclimated for 2 h, stimulated for 5 min (20 Hz, 5 ms pulses), allowed to rest for 20 min, and then removed and blood collected. These three time points were separated by 2-week intervals to avoid confounding results from the stressful process of blood collection, and to allow animals time to recover from blood loss between collections. Plasma was collected by puncturing the submandibular vein with a lancet and collecting whole blood into EDTA-treated vials. For catecholamines (epinephrine and norepinephrine), EGTA-glutathione solution was prepared according to Vanderbilt University Hormone Assay and Analytical Services Core specifications to be used as a preservative. Briefly, 4.5 g EGTA and 3.0 g glutathione was dissolved in 50 mL dIH20 (pH of 6.0–7.4). The EGTA-glutathione solution was added to catecholamine tubes immediately upon blood collection at a concentration of 1:50. Blood was spun down at 14,000*g* for 15 min at 4 °C to isolate plasma. Plasma was removed from the top layer, flash frozen, and stored at − 80 °C until being sent to the Vanderbilt Hormone and Analytics Core for hormone analysis. ACTH and corticosterone levels were measured using radioimunnoassays via a double antibody procedure, and catecholamines norephinephrine and epinephrine using HPLC via electrochemical detection. Statistical significance was measured using a repeated-measures Two-way ANOVA with a Sidak correction for multiple comparisons.

### Food choice assay using chow and high-fat chow paired with optogenetic stimulation

Male and female vGlut2-Cre^+/−^ littermates were stereotaxically injected with rAAV-flex-ChR2-EYFP or rAAV-flex-GFP into the BF and implanted with fiber optics over the LHb to stimulate BF terminals. Mice were fasted overnight and placed in a large rectangular arena (25in x 17in) where they were allowed to acclimate for 5 min. On one side of an arena, chow was placed securely in one corner, while high fat chow was placed securely in the adjacent corner. Using the open source.

Bonsai^68^, photostimulation was limited to a ROI along the food side of the arena, just large enough to photostimulate the mouse while consuming or interacting with food. Mice were video recorded and food intake was recorded for a total of 20 min. Average food intake for both the chow and high fat chow was calculated for both ChR2-EYFP and GFP mice and were compared using Two-way ANOVA. Time spent in the stimulation side of the arena, as well as the time spent interacting with either food choice were calculated post-hoc using Optimouse in MATLAB^69^, and were compared using a Two-way ANOVA.

## Supplementary Information

Supplementary Legends.

Supplementary Figure 1.

Supplementary Figure 2.

Supplementary Figure 3.

Supplementary Figure 4.

Supplementary Figure 5.

Supplementary Figure 6.

Supplementary Figure 7.

Supplementary Figure 8.

Supplementary Figure 9.

Supplementary Figure 10.

Supplementary Figure 11.

## Data Availability

The datasets generated and/or analyzed during the current study are available from the corresponding author upon reasonable request.
