# Supplementary material for: Activation of basal forebrain-to-lateral habenula circuitry drives reflexive aversion and suppresses feeding behavior
Source: Sci Rep. 2022 Dec 21;12:22044. doi: 10.1038/s41598-022-26306-8 (PMC9772215; doi:10.1038/s41598-022-26306-8)
Supplement: Supplementary file 1 — Supplementary Legends. [file 41598_2022_26306_MOESM1_ESM.docx]

**Activation of basal forebrain-to-lateral habenula circuitry drives reflexive aversion and suppresses feeding behavior**

Jessica L. Swanson, Joshua Ortiz-Guzman, Snigdha Srivastava, Pey-Shyuan Chin, Sean W. Dooling, Elizabeth Hanson Moss, Mikhail Y. Kochukov, Patrick J. Hunt, Jay M. Patel, Brandon T. Pekarek, Qingchun Tong, & Benjamin R. Arenkiel

**Supplemental Figure Legends**

**Supplemental Figure 1: The glutamatergic basal forebrain innervates numerous brain regions in addition to the lateral habenula.**

1. Piriform Cortex at Bregma 1.10.
2. Hypothalamic region encompassing the LHA, DMH, and VMH at Bregma -1.70.
3. Premammillary nucleus at Bregma -2.70.
4. Ventral tegmental area (more anterior portion) at Bregma -3.08. Scale bar= 200 μm.
5. Periaqueductal Gray at Bregma -3.08.
6. Ventral Tegmental area (posterior portion) and interpeduncular nucleus at Bregma -3.52.

**Supplemental Figure 2: Fiber optic placement for animals recorded using fiber photometry.**

1. Unilateral fiber optic targeting for BF axon terminal fiber photometry. Each symbol represents an individual animal.
2. Unilateral fiber optic targeting of LHb soma targeted using AAV1-Cre targeted to the BF. Each symbol represents an individual animal.

**Supplemental Figure 3: Enlarged heat maps of glutamatergic BF terminal fiber photometry.** Enlarged heat maps from Fig. 2c. Each row on the heat map represents an individual odor presentation. Each odor was presented in replicates of 10. Individual mice are separated by red lines. Heat maps generated using MATLAB (version R2019a; https://www.mathworks.com/products/matlab.html).

**Supplemental Figure 4: Glutamatergic BF terminals in the LHb respond to diverse odor stimuli.**

1. Average z-score dF/F traces of vGlut2^BF🡪LHb^ axon terminals (in black) across biological replicates (n=12). Grey outline represents 95% CI. Pink box represents 2 s odor delivery. Heat map shows each presentation of a given odor (10 replicates per odor) across all 12 mice. Yellow= 1 (maximum activity), dark blue=0 (minimum activity). Heat maps generated using MATLAB (version R2019a; https://www.mathworks.com/products/matlab.html).
2. Mean odor response of BF Terminals recorded using fiber photometry. Error bars represent SEM. n= 12. Mean z-score dF/F odor responses: Mineral oil= 0.19±0.22 (p=0.4130), +Limonene= 0.62±0.34 (p=0.0936), -Limonene= 0.89±0.19 (p=0.0008), Rose Oil= 0.74±0.30 (p=0.0291), Peanut butter= 0.22±0.29 (p=0.4592).
3. Mean odor response area under the curve (AUC) of BF Terminals recorded using fiber photometry. Error bars represent SEM. n= 12. Mean AUC of z-score dF/F odor responses: Mineral oil= 78.61±80.24 (p=0.3483), +Limonene= 220.9±124.3 (p=0.1032), -Limonene= 327.6±70.69 (p=0.0007), Rose Oil= 270.4±110.4 (p=0.0323), Peanut butter= 84.52±105.9 (p=0.4418).

**Supplemental Figure 5: Enlarged heat maps of LHb soma receiving BF input fiber photometry.** Enlarged heat maps from Fig. 2g. Each row on the heat map represents an individual odor presentation. Each odor was presented in replicates of 10. Individual mice are separated by red lines. Heat maps generated using MATLAB (version R2019a; https://www.mathworks.com/products/matlab.html).

**Supplemental Figure 6: LHb cells receiving BF input respond to diverse odor stimuli.**

1. Average z-score dF/F traces of LHb cells receiving BF input (in black) across biological replicates (n=6). Grey outline represents 95% CI. Pink box represents 2 s odor delivery. Heat map shows each presentation of a given odor (10 replicates) across all 6 mice. Yellow= 1 (maximum activity), dark blue=0 (minimum activity). Heat maps generated using MATLAB (version R2019a; https://www.mathworks.com/products/matlab.html).
2. Mean odor response of LHb cells receiving BF input recorded using fiber photometry. Error bars represent SEM. n=6. Mean z-score dF/F odor responses: Mineral oil: -0.14±0.34 (p=0.6912), +Limonene= 1.00±0.24 (p=0.0086), -Limonene= 1.98±0.075 (p=<0.0001), Rose Oil= 1.61±0.13 (p=<0.0001), Peanut butter= 0.83±0.24 (p=0.0165).
3. Mean odor response area under the curve (AUC) of LHb cells receiving BF input recorded using fiber photometry. Error bars represent SEM. n=6. Mean AUC of z-score dF/F odor responses: Mineral oil= -51.10±123.3 (p=0.6957), +Limonene= 360.5±89.06 (p=0.0098), -Limonene= 728.9±27.90 (p=<0.0001), Rose Oil= 577.5±49.84 (p=<0.0001), Peanut butter= 277.7±99.02 (p=0.0378).

**Supplemental Figure 7: Fiber optic targeting for optogenetic stimulation of glutamatergic BF terminals in the LHb.** Implants were bilaterally targeted above the LHb at a 15°. LHb is outlined in black next to the MHb filled in light grey. Each color set represents an individual animal where circles are for ChR2-EYFP animals and X’s are for GFP controls. n=7.

**Supplemental Figure 8: Optogenetic implant animals can perform contextual fear conditioning as normal in the absence of stimulation.**

1. Experimental paradigm for contextual fear conditioning.
2. Average percent time spent freezing 2 and 24 hours post training as compared to in the naïve state. Error bars represent SEM. Statistical significance determined using repeated measures two-way ANOVA with Sidak correction for multiple comparisons. For **GFP controls**: Naïve (pre-shock control)= 0.00±0.00%, 2-hrs post-shock= 13.08±2.33% (p=0.0047), 24-hrs post shock= 20.07±3.20% (p=<0.0001). GFP 2hrs vs. 24hrs: p=0.1878. For **ChR2-EYFP animals**: Naïve (pre-shock control)= 0.82±0.24%, 2-hrs post-shock= 17.63±4.57% (p=0.0002), 24-hrs post-shock= 20.10±1.62% (p=<0.0001). ChR2 2hrs vs. 24hrs: p=0.8516.

**Supplemental Figure 9: Optogenetic inhibition of glutamatergic BF terminals prevents synaptic transmission to the LHb.**

1. Experimental setup for whole cell voltage clamp electrophysiological recordings, in which a 1:1 mixture of Cre-dependent ArchT-GFP and ChR2-EYFP was targeted to the BF. Whole cell recordings were performed in the LHb while stimulating and/or inhibiting BF terminals expressing ChR2 and ArchT. 470 nm light stimulation was used activate BF terminals in the LHb via ChR2, while 565 nm light was used to inhibit BF terminals via ArchT.
2. Representative viral targeting of Cre-dependent ArchT-GFP and ChR2-EYFP mixture to the BF (Bregma 0.98). Scale bar= 200 μm.
3. ArchT-GFP/ChR2-EYFP-expressing fibers from vGlut2^BF^ neurons terminating in the LHb. Scale bar= 300 μm. ii) Zoomed-in inset of left LHb. MHb= medial habenula. Scale bar= 200 μm.
4. Representative trace of a LHb cell throughout a recording session. First, 470nm light pulses were used to activate presynaptic ChR2, eliciting a postsynaptic LHb response. Then, continuous 565nm light stimulation was used to activate presynaptic ArchT, and 470nm pulses were delivered simultaneously to test whether ArchT photoinhibition of BF terminals suppressed ChR2-evoked responses in LHb target cells. Finally, 565nm light was removed, and 470nm light pulses were used to reversibility activate presynaptic ChR2, eliciting a postsynaptic LHb response.
5. Average amplitude of LHb postsynaptic response to presynaptic BF ChR2 or ChR2/ArchT photostimulation. Each data point represents an average across 10 sweeps. Average ChR2-evoked response (470 nm alone)= 57.67±32.99 pA. Average ChR2 + ArchT-evoked response (565 nm + 470 nm)= 2.33±1.45 pA. Average ChR2-evoked response post-inhibition (470 nm alone)= 63.33±37.16 pA. n=3.
6. Average amplitude of LHb postsynaptic response normalized to the ChR2-evoked response upon 470 nm light stimulation (represented as a percent). Average response of ChR2 + ArchT stimulation (both 565 and 470 nm)= 3.38±1.78%. Average response of ChR2 stimulation post-inhibition (470 nm alone)= 107.80±1.80%. Mean percent amplitude responses compared using a one-way ANOVA with a Bonferonni correction for multiple comparisons. ChR2 stim vs. ChR2 + ArchT stim: p=<0.0001. ChR2 stim vs. ChR2 stim (post): p=0.0895. ChR2 + ArchT stim vs. ChR2 stim (post): p=<0.0001. n=3.

**Supplemental Figure 10: Optogenetic inhibition of the LHb does not affect feeding or aversive behaviors.**

1. Experimental setup for *in vivo* optogenetic behavior, in which vGlut2^BF^ terminals in the LHb are inhibited.
2. Representative images showing viral targeting of Cre-dependent ArchT-GFP to the BF and fiber optic targeting to the LHb. Basal forebrain image taken at Bregma 0.50. Lateral habenula image taken at Bregma -1.46.
3. Food intake of fasted ArchT-GFP animals during 5 minute bouts without photoinhibition (black line), or with photoinhibition (green line). Solid symbols represent average values, while hollow/transparent symbols represent individual biological replicates. Error bars represent SEM. Statistical significance calculated using a repeated measures two-way ANOVA with a Sidak correction for multiple comparisons. n=8. At 5 min: Non-stimulated= 0.178±0.017g while stimulated= 0.088±0.025g (p= 0.0022). At 10 min: Non-stimulated= 0.104±0.017g while stimulated= 0.099±0.020g (p=0.9993). At 15 min: non-stimulated= 0.080±0.009g while stimulated= 0.070±0.014g (p=0.9902). At 20 min: non-stimulated= 0.114±0.018g while stimulated= 0.073±0.014g (p=0.3384).
4. Experimental setup for real-time place preference assay with optogenetic stimulation.
5. Heat maps showing movement of representative GFP control and ArchT-GFP mice during the real-time place preference assay in which mice were stimulated on the right side of the chamber. Heat maps generated using Noldus EthoVision software (XT 16; https://www.noldus.com/ethovision-xt).
6. Average percent time GFP controls and ArchT-GFP expressing animals spent in non-stimulation or stimulation sides of the arena during a 20 min experiment. Error bars represent SEM. Statistical significance determined using Binomial test for proportion with Bonferroni correction; null hypothesis=50%. n=7. For GFP controls, they spent 47.68±1.98% of their time in the non-stim side and 51.32±1.97% in the stim side, p=0.7644. For ArchT: 47.47±5.41% time in non-stim side, 50.69±5.76% in stim side, p=0.7644.

**Supplemental Figure 11: Fiber optic targeting for optogenetic inhibition of glutamatergic BF terminals in the LHb.** Implants were bilaterally targeted above the LHb at a 15°. LHb is outlined in black next to the MHb filled in light grey. Each color set represents an individual animal. n=8.
