# Supplementary figures and images for: Activation of basal forebrain-to-lateral habenula circuitry drives reflexive aversion and suppresses feeding behavior
Source: Sci Rep. 2022 Dec 21;12:22044. doi: 10.1038/s41598-022-26306-8 (PMC9772215; doi:10.1038/s41598-022-26306-8)

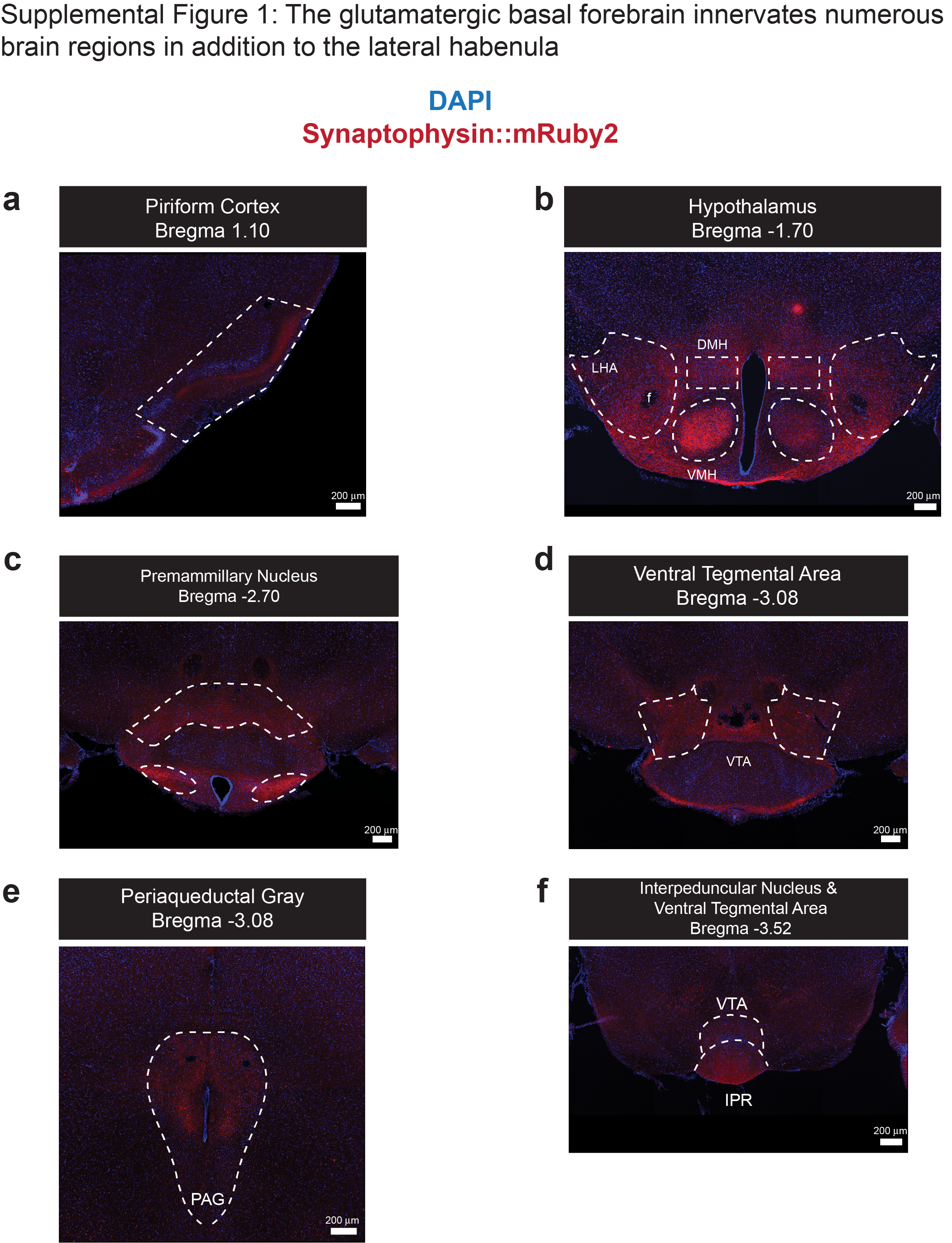

Supplement: Supplementary file 2 — Supplementary Figure 1. [file 41598_2022_26306_MOESM2_ESM.png]

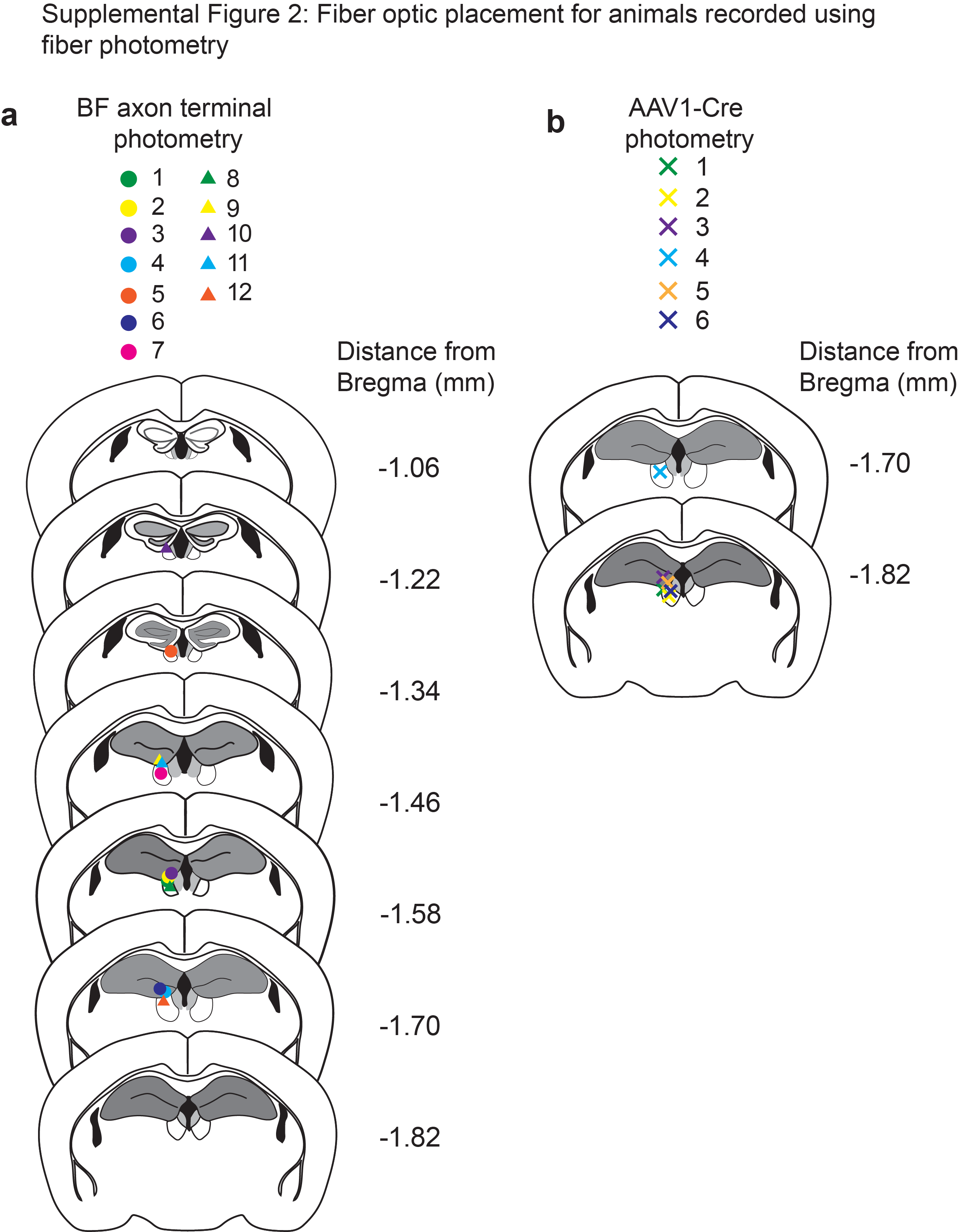

Supplement: Supplementary file 3 — Supplementary Figure 2. [file 41598_2022_26306_MOESM3_ESM.png]

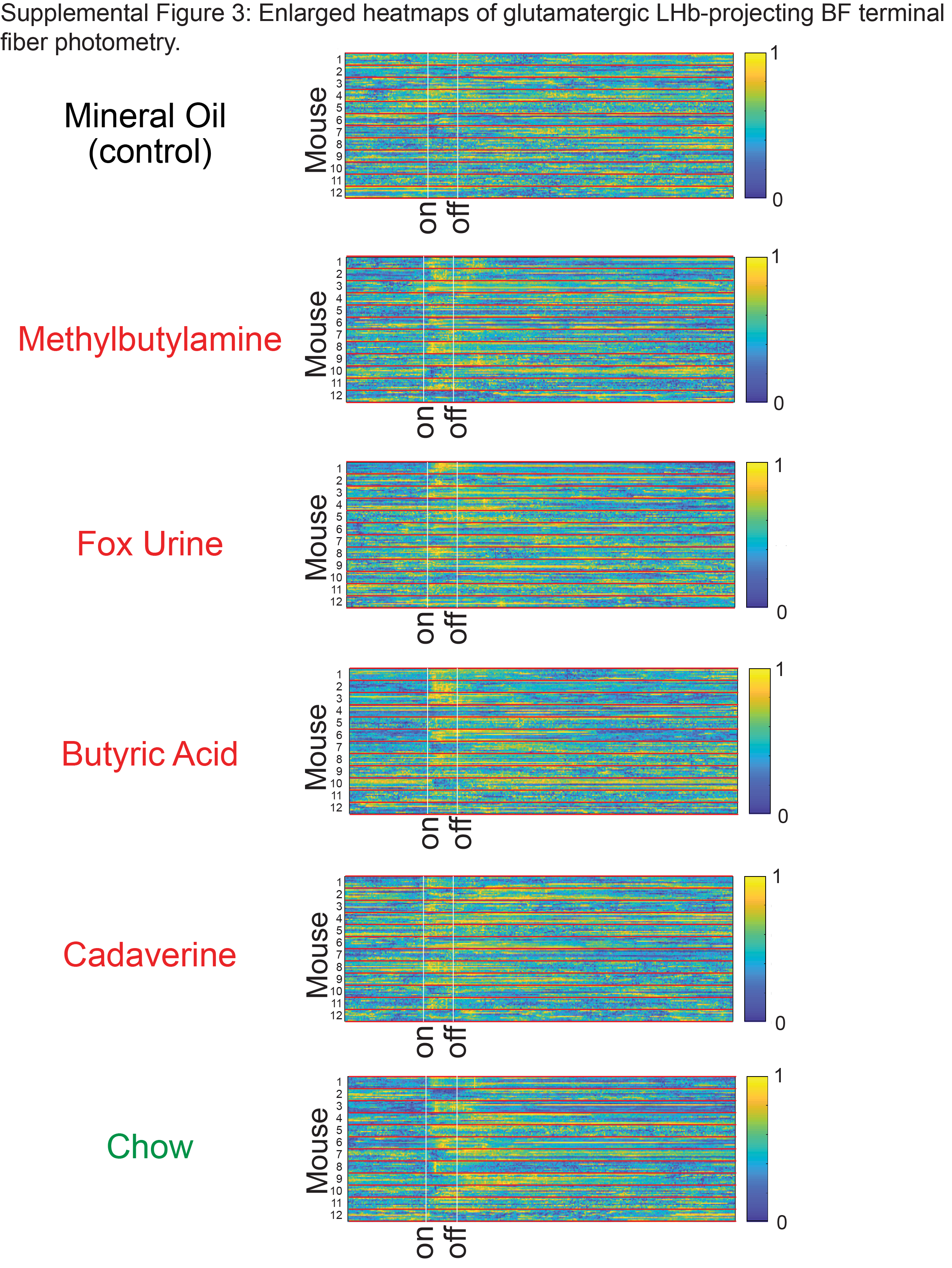

Supplement: Supplementary file 4 — Supplementary Figure 3. [file 41598_2022_26306_MOESM4_ESM.png]

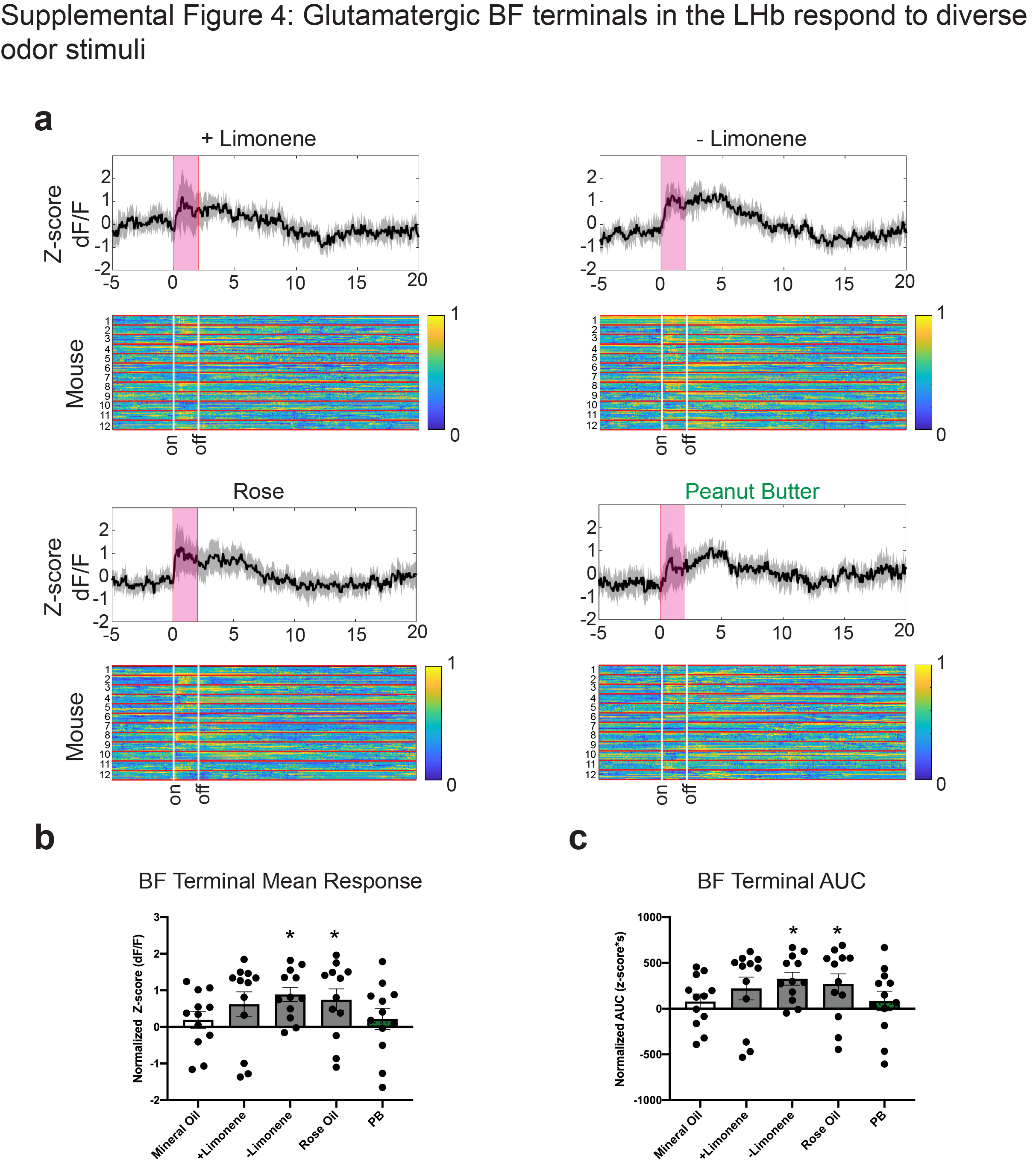

Supplement: Supplementary file 5 — Supplementary Figure 4. [file 41598_2022_26306_MOESM5_ESM.png]

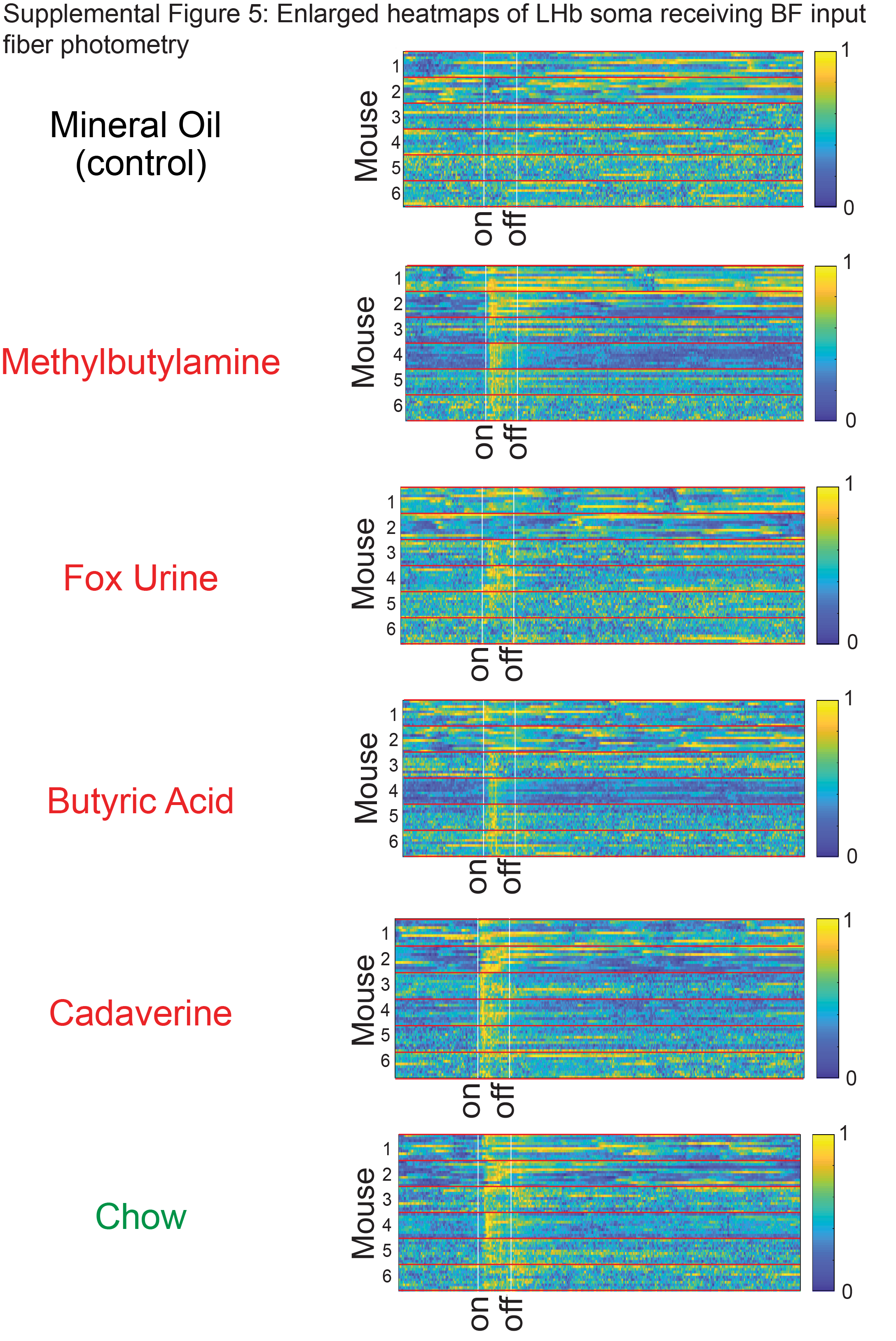

Supplement: Supplementary file 6 — Supplementary Figure 5. [file 41598_2022_26306_MOESM6_ESM.png]

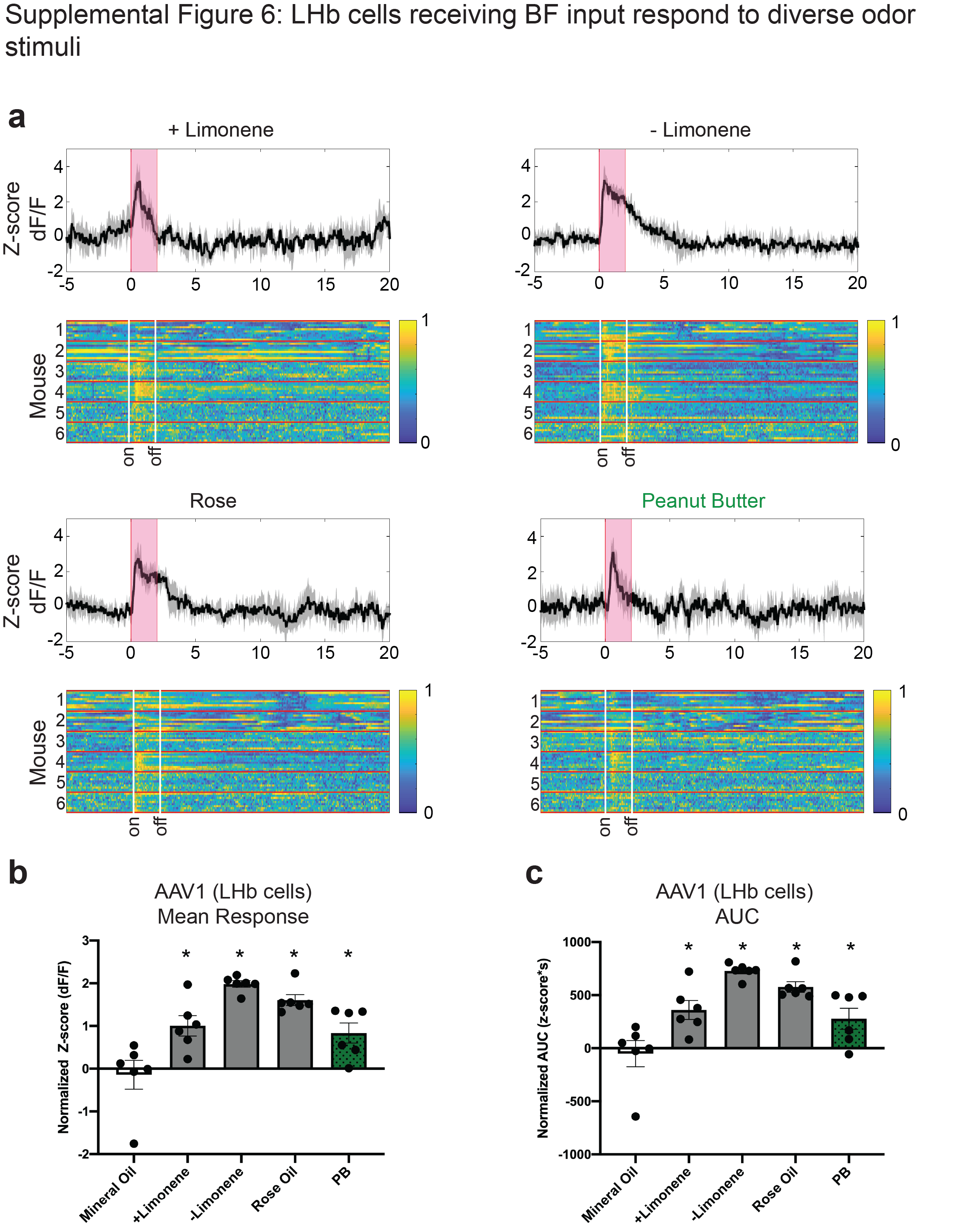

Supplement: Supplementary file 7 — Supplementary Figure 6. [file 41598_2022_26306_MOESM7_ESM.png]

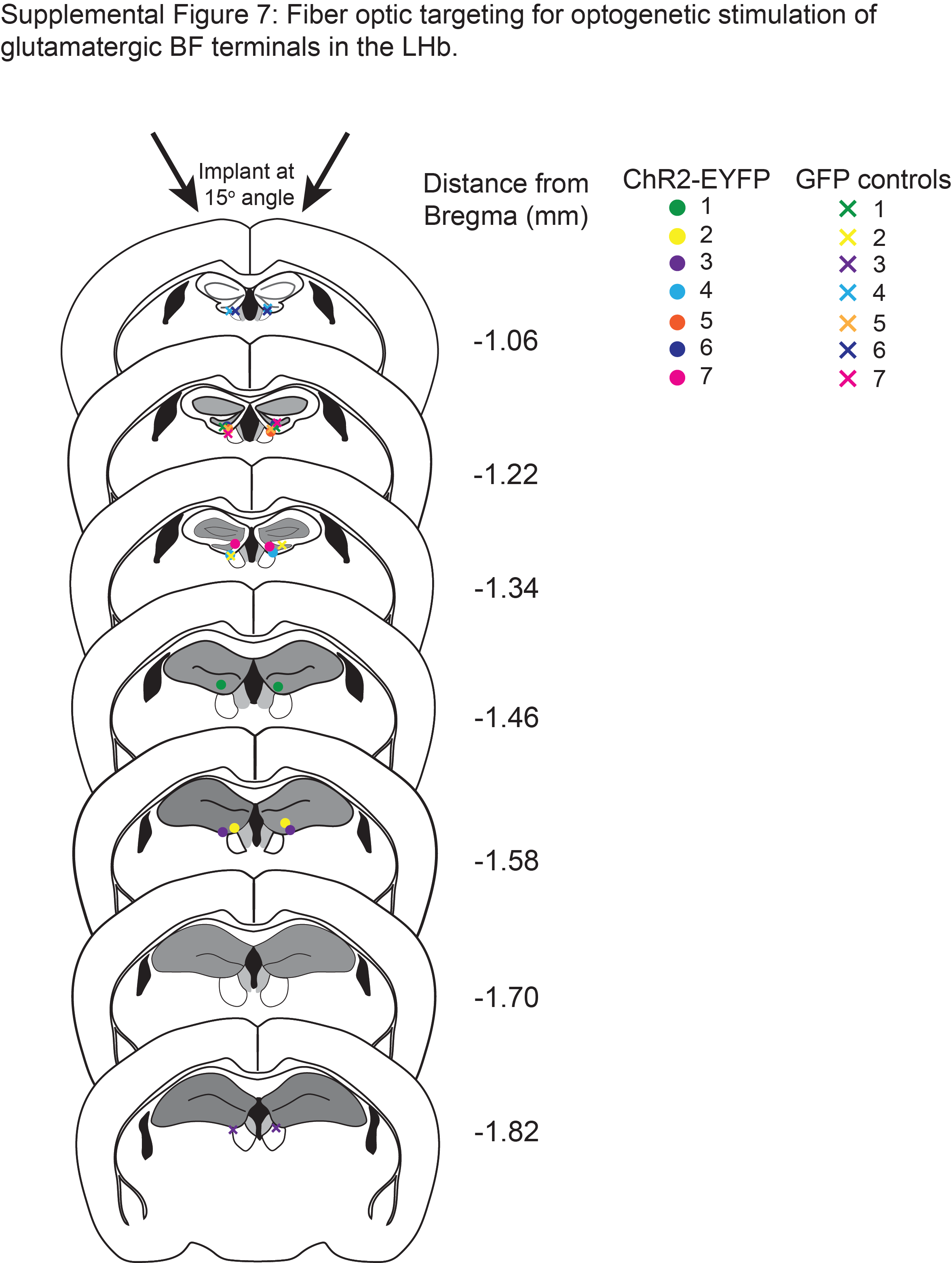

Supplement: Supplementary file 8 — Supplementary Figure 7. [file 41598_2022_26306_MOESM8_ESM.png]

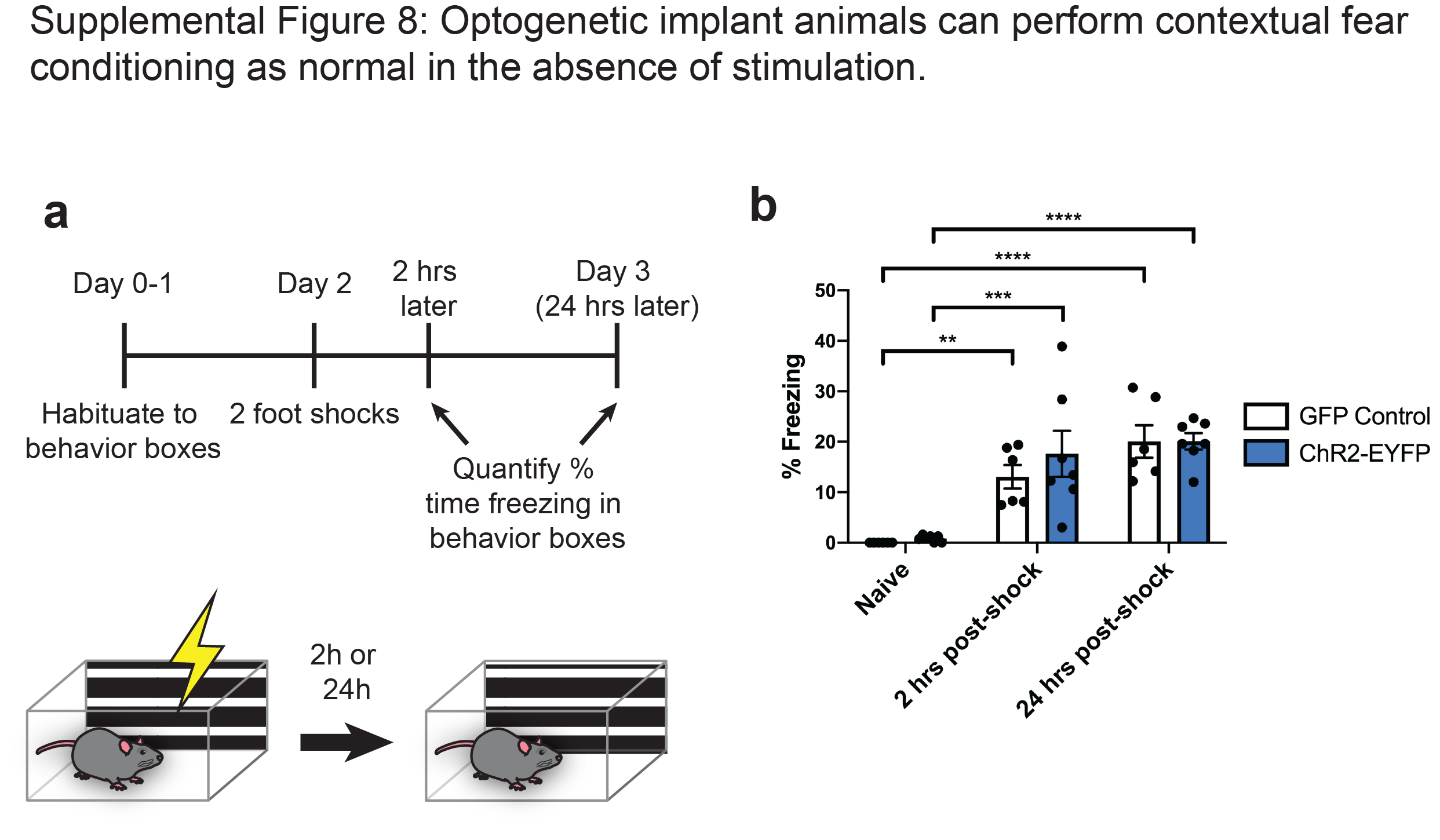

Supplement: Supplementary file 9 — Supplementary Figure 8. [file 41598_2022_26306_MOESM9_ESM.png]

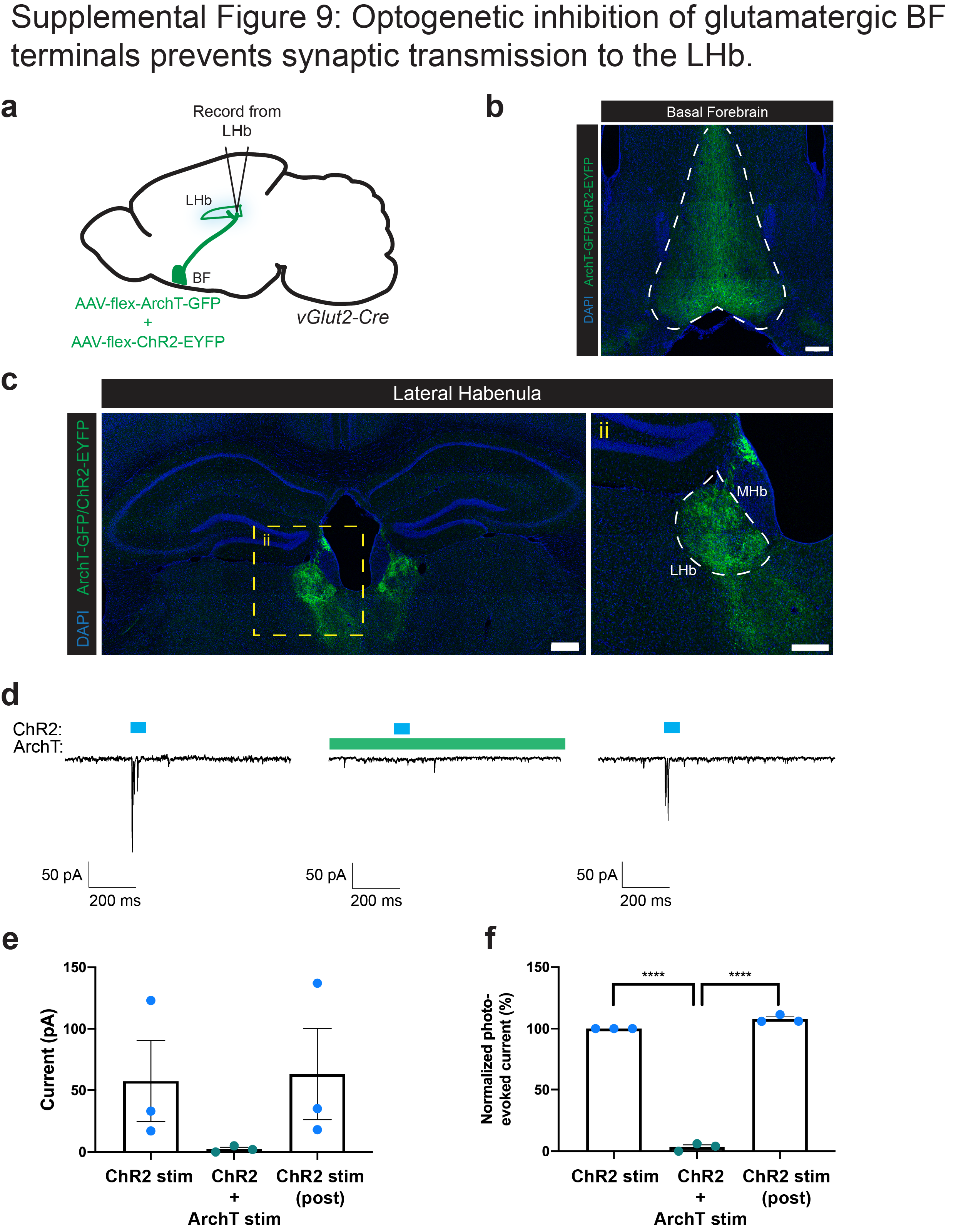

Supplement: Supplementary file 10 — Supplementary Figure 9. [file 41598_2022_26306_MOESM10_ESM.png]

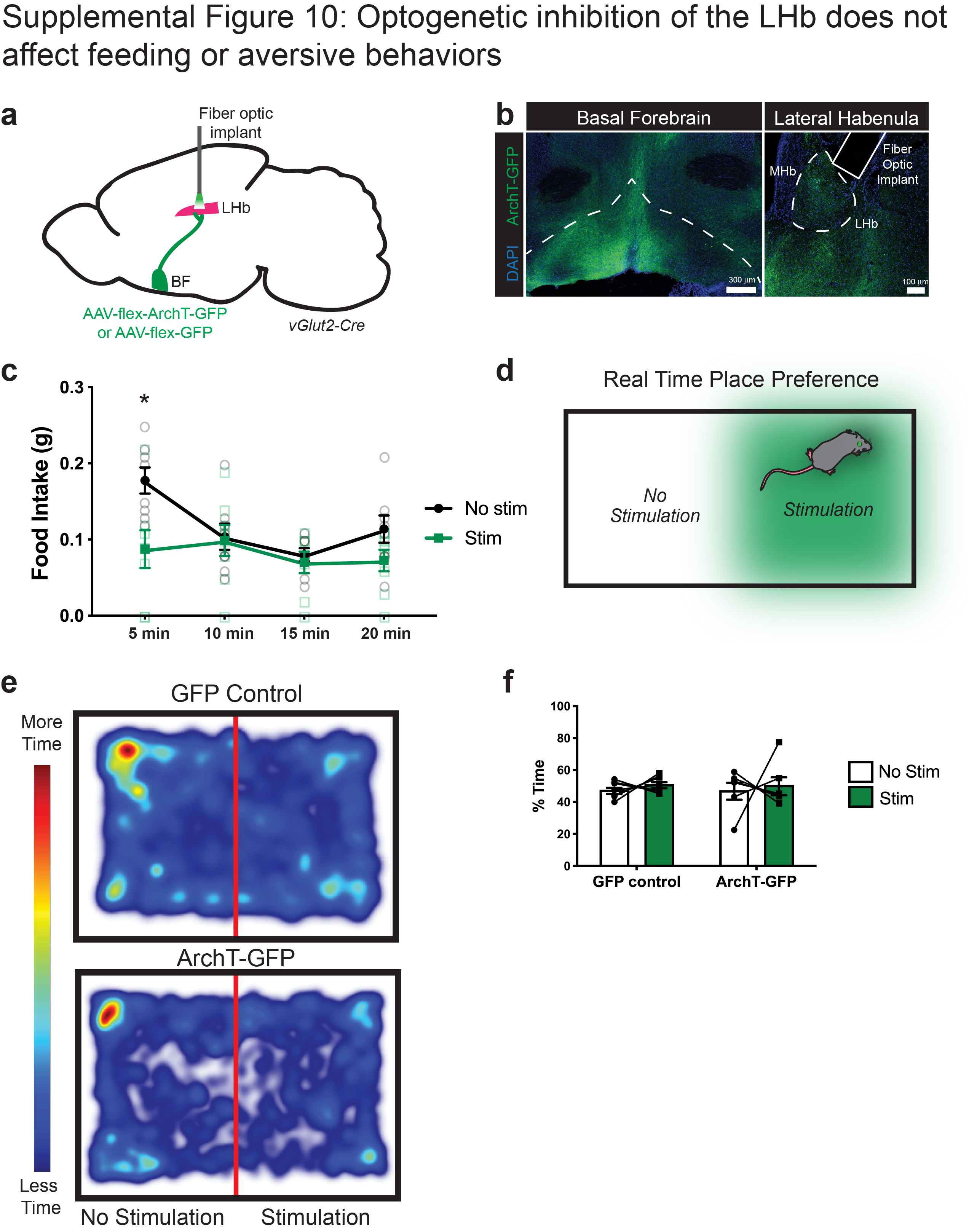

Supplement: Supplementary file 11 — Supplementary Figure 10. [file 41598_2022_26306_MOESM11_ESM.png]

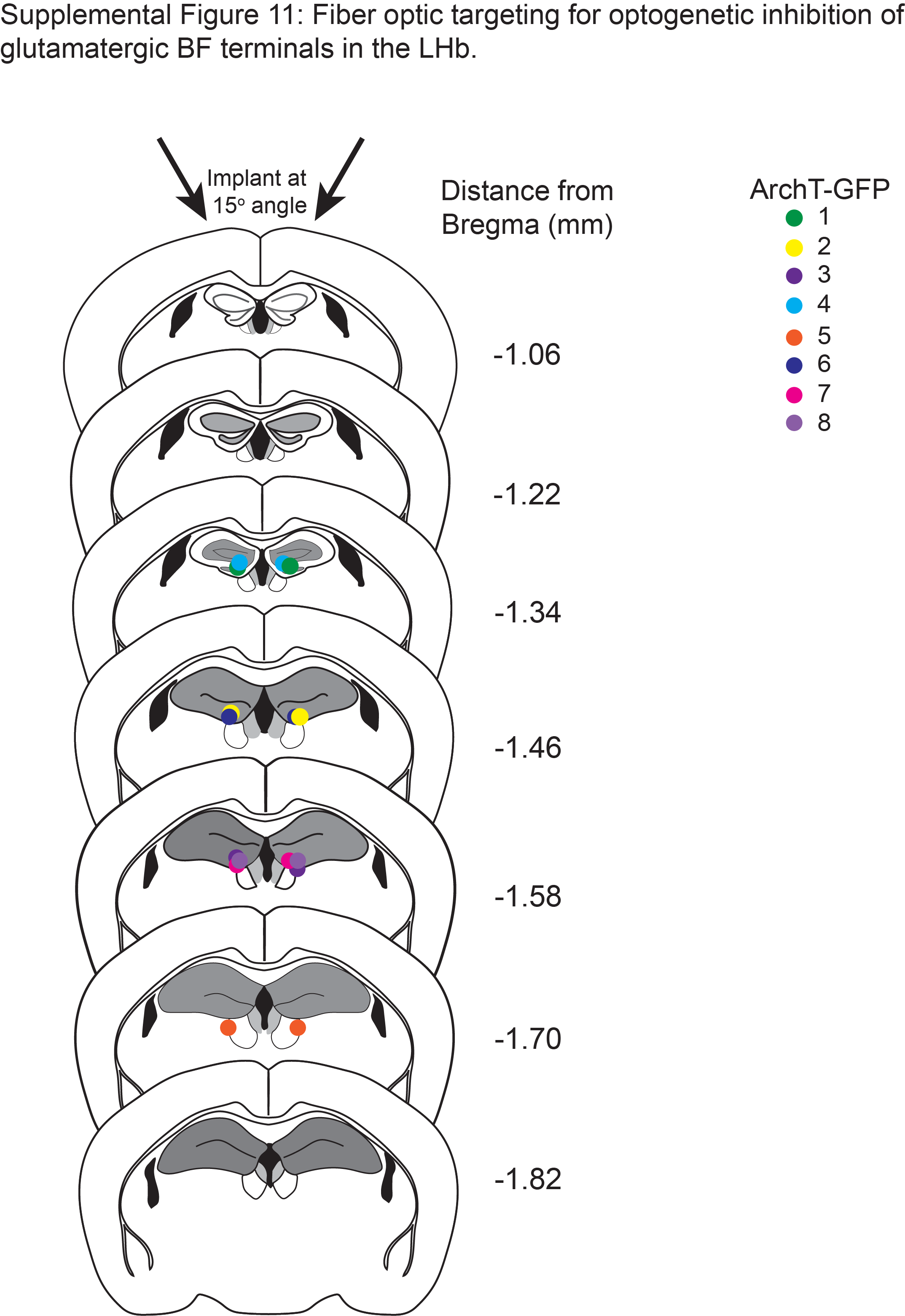

Supplement: Supplementary file 12 — Supplementary Figure 11. [file 41598_2022_26306_MOESM12_ESM.png]
